# Supplementary material for: Seasonal Proteome Variations in Orbicella faveolata Reveal Molecular Thermal Stress Adaptations
Source: Proteomes. 2024 Jul 10;12(3):20. doi: 10.3390/proteomes12030020 (PMC11270422; doi:10.3390/proteomes12030020)
Supplement: Supplementary file 1 [file proteomes-12-00020-s001.zip › O.f. Supp. Fig. S1 05.23.24.pdf]

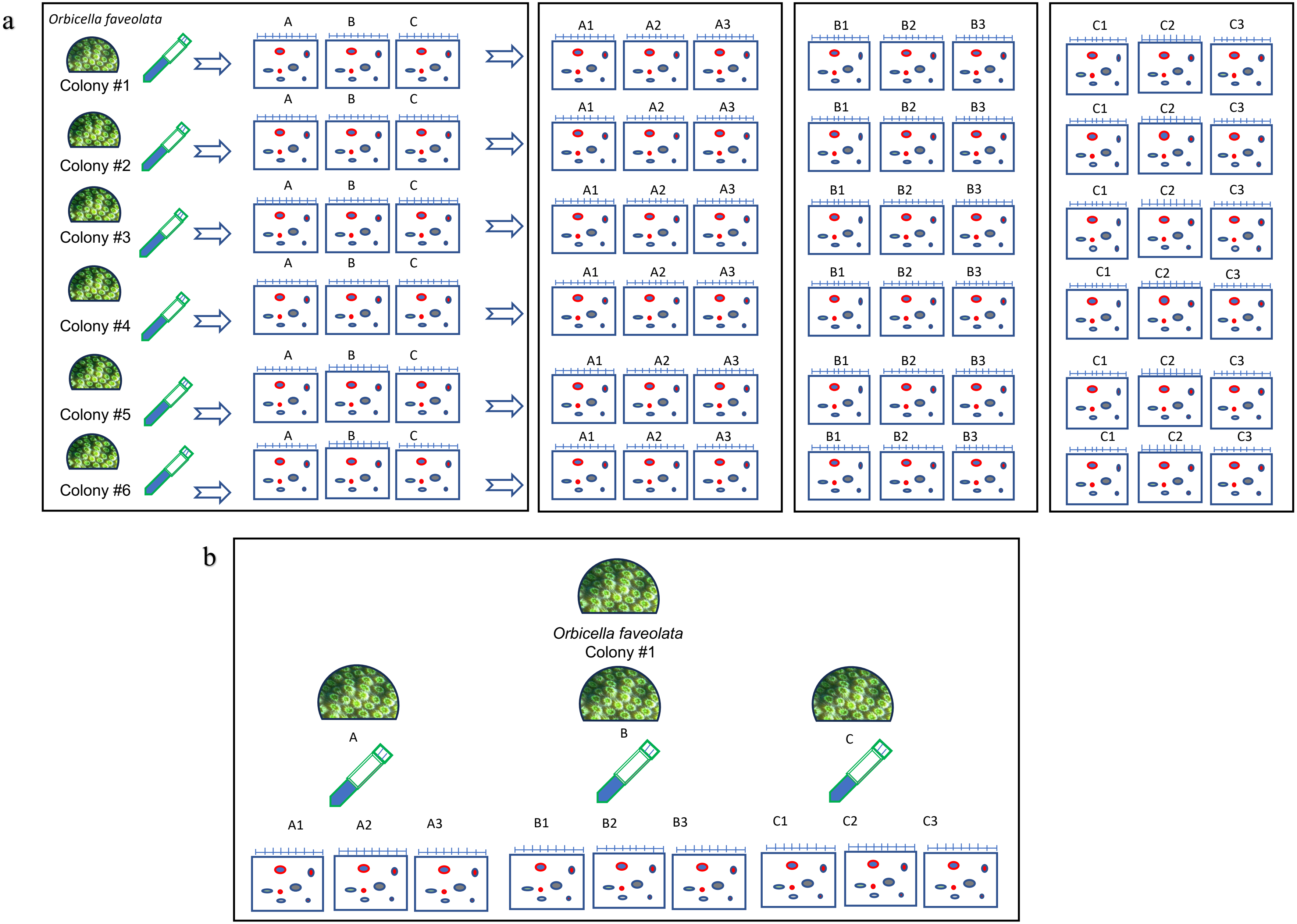

Supplementary Figure S1. Biological replicates, and validation of presented proteomic analysis comparison of the three independent *Orbicella faveolata* were performed using strategy described by the scheme.
a: Biological replicates derived from independent samples helped capture random biological variation. For example, three biological replicates (A, B, and C) were collected from three independent *Orbicella faveolata*. ***b:*** Each of these biological replicates was run in three technical replicates (A1, A2, A3; B1, B2, B3; C1, C2, C3) in one 2D-GE protein separation (B). Technical replicates help identify variation in technique. For example, lysate derived from *Orbicella faveolata* were loaded three times (A1, A2, A3) on each 2D gel then run and measured independently to help identifying variation in technique (B).
