# Supplementary material for: Seasonal Proteome Variations in Orbicella faveolata Reveal Molecular Thermal Stress Adaptations
Source: Proteomes. 2024 Jul 10;12(3):20. doi: 10.3390/proteomes12030020 (PMC11270422; doi:10.3390/proteomes12030020)
Supplement: Supplementary file 1 [file proteomes-12-00020-s001.zip › O.f. Supp. Table S1. 02.10.24.pdf]

Supplementary Table S1. Statistical analysis of proteoforms data from *Orbicella faveolata*.

Question: Will the temperature affect the protein abundance in *Orbicella faveolata* colonies collected during cold and warm seasons during 2014-2015 in Puerto Rico?

H0: Temperature does not affect protein abundance in *Orbicella faveolata* during both seasons

H1: There are significant differences in the proteins expressed in *Orbicella faveolata* during the two seasons.

1. Analysis of variance

Variable	N	R^2	Adj R^2	CV	
Protein expression	336	0.47	0.15	5.19	
Analysis of variance table (Partial SS)					
S.V.	SS	df	MS	F	p-value
Model	398.94	126	3.17	1.46	0.0079
PROT_EXP	398.94	126	3.17	1.46	0.0079
Error	453.22	209	2.17		
Total	852.06	335			

p value < 0.05 reject the null hypothesis

There are significant protein expression levels of which change in response to the season temperature.

2. Shapiro Wilks to identify if the data deviates from a comparable normal distribution

**Shapiro-Wilk (modified)**					
Variable	n	Mean	S.D.	W*	p(one tail)
RES protein expression	336	76.86	62.27	0.89	<0.0001

For significant levels of expression $p < 0.05$, Shapiro–Wilk ($p < 0.0001$) and Levene’s tests were used to determinewhether all proteins expressed in *Orbicella faveolata* were normally distributed and of homogeneous variance, respectively.

Continuation Supplementary Table S1.

3. One-Sample t-Test

Mean value under the null hypothesis: 0							
Variable	n	Mean	SD	LL (95)	UL (95)	T	p (Two tails)
Protein expression	336	76.86	62.27	70.17	83.54	22.62	<0.0001

4. T-Test for independent samples (protein expression vs. season temperature) 2014 year

Class	Variable	Group 1	Group 2	n (1)	n (2)	Mean (1)	Mean (2)
Season	protein expression	{26.60}	{26.70}	42	42	78.82	76.75
Season	protein expression	{26.60}	{29.80}	42	42	78.82	75.48
Season	protein expression	{26.60}	{30.09}	42	42	78.82	60.34
Season	protein expression	{26.70}	{29.80}	42	42	76.75	75.48
Season	protein expression	{26.70}	{30.09}	42	42	76.75	60.34
Season	protein expression	{29.80}	{30.09}	42	42	75.48	60.34

Mean (1)-Mean (2)	LL (95)	UL (95)	pVarHom	T	p-value	Test
2.07	-23.79	27.93	0.4669	0.16	0.8738	Two tails
3.34	-22.43	29.12	0,4915	0.26	0.7970	Two tails
18.48	-5.16	42.12	0.7004	1.55	0.1238	Two tails
1.27	-25.94	28.48	0.9683	0.09	0.9262	Two tails
16.41	-8.79	41.61	0.2668	1.30	0.1989	Two tails
15.14	-9.97	40.25	0.2842	1.20	0.2339	Two tails

1. T-Test for independent samples (protein expression vs. season temperature) 2015 year

Class	Variable	Group 1	Group 2	n (1)	n (2)	Mean (1)	Mean (2)
Season	protein expression	{26.90}	{26.96}	42	42	87.16	89.49
Season	protein expression	{26.90}	{29.80}	42	42	87.16	80.12
Season	protein expression	{26.90}	{30.15}	42	42	87.16	66.69
Season	protein expression	{26.96}	{29.80}	42	42	89.49	80.12
Season	protein expression	{26.96}	{30.15}	42	42	89.49	66.69
Season	protein expression	{29.80}	{30.15}	42	42	80.12	66.69

Mean (1)-Mean (2)	LL (95)	UL (95)	pVarHom	T	p-value	Test
-2.33	-31.55	26.89	0.4534	-0.16	0.8742	Two tails
7.04	-22.93	37.01	0.6910	0.47	0.6414	Two tails
20.47	-8.25	49.19	0.3154	1.42	0.1600	Two tails
9.37	-18.87	37.62	0.7241	0.66	0.5109	Two tails
22.80	-4.11	49.72	0.7985	1.69	0.0957	Two tails
13.43	-14.30	41.16	0.5432	0.96	0.3381	Two tails
