# Supplementary material for: Seasonal Proteome Variations in Orbicella faveolata Reveal Molecular Thermal Stress Adaptations
Source: Proteomes. 2024 Jul 10;12(3):20. doi: 10.3390/proteomes12030020 (PMC11270422; doi:10.3390/proteomes12030020)
Supplement: Supplementary file 1 [file proteomes-12-00020-s001.zip › O.f. Supp. Table S2. DeltaCt 02.12.24.pdf]

Supplementary Table S2: Statistical analysis from the RT-PCR results in *Orbicella faveolata*

Formulas:

(Hellemans et al. 2008)

Abbreviations:

EN: Inner-shelf Enrique

SC: Mid-shelf San Cristobal

$$Ratio = \frac{(E_{target})^{\Delta Ct_{target}}}{(E_{reference})^{\Delta Ct_{reference}}} \quad Equation\ 1$$

Whereas $\Delta Ct_{target} = Ct_{control} - Ct_{treatment}$ and $\Delta Ct_{reference} = Ct_{control} - Ct_{treatment}$

$$Ratio = 2^{-\Delta\Delta Ct} \quad Equation\ 2$$

Whereas $\Delta\Delta Ct = \Delta Ct_{reference} - \Delta Ct_{target}$

Reference gene with Ct values for *Orbicella faveolata* colonies in Enrique and San Cristobal reefs

EN		A1	A2
CS	Actin	23.23	21.40
	Actin	22.96	22.30
	Actin	23.24	25.10
WS	Actin	16.20	17.19
	Actin	14.20	15.14
	Actin	13.60	13.12

Inner-shelf reef Enrique												
**Season**	**Sample**	**Gene**	**CT (B1)**	**B1-A1**	**Average ΔCT**	**ΔΔCT**	**2^-(ΔΔCT)**	**log₁₀2^-(ΔΔCT)**	**Mean log₁₀2^-(ΔΔCT)**	**Mean**	**STD**	**CV%**
CS	Control	COI	6.22	-17.01	-16.9	-0.13	1.1	0.0	0.0	6.3	0.9	13.6
			5.24	-17.72		-0.84	1.8	0.3				
			7.33	-15.91		0.97	0.5	-0.3				
WS	Treated		33.01	16.81		33.69	0.0	-10.1	-10.9	34.1	0.9	2.6
			35.15	20.95		37.83	0.0	-11.4				
			34.14	20.54		37.42	0.0	-11.3				
CS	Control	Green fluorescent	6.30	-16.93	-16.8	-0.15	1.1	0.0	0.0	6.4	0.2	2.7
			6.20	-16.76		0.02	1.0	0.0				
			6.60	-16.64		0.14	0.9	0.0				
WS	Treated		18.33	2.13		18.91	0.0	-5.7	-6.1	18.2	0.7	4.0
			17.25	3.05		19.83	0.0	-6.0				
			19.01	5.41		22.19	0.0	-6.7				
CS	Control	Beta tubulin	33.01	9.78	11.0	-1.18	2.3	0.4	0.0	34.1	0.9	2.6
			35.15	12.19		1.23	0.4	-0.4				
			34.14	10.90		-0.06	1.0	0.0				
WS	Treated		10.12	-6.08		-17.04	134445.9	5.1	4.4	10.9	0.6	5.1
			11.00	-3.20		-14.16	18263.4	4.3				
			11.45	-2.15		-13.11	8820.6	3.9				
CS	Control	Cysteine rich protein	5.53	-17.70	-11.7	-6.04	65.6	1.8	0.0	5.8	0.4	7.0
			6.35	-16.61		-4.95	30.8	1.5				
			5.45	-0.68		10.98	0.0	-3.3				
WS	Treated		23.43	7.23		18.89	0.0	-5.7	-5.9	22.5	0.8	3.7
			21.42	7.22		18.88	0.0	-5.7				
			22.56	8.96		20.62	0.0	-6.2				
CS	Control	α carbonic anhydrase	31.21	7.98	9.2	-1.18	2.3	0.4	0.0	32.3	0.8	2.6
			33.24	10.28		1.12	0.5	-0.3				
			32.45	9.21		0.05	1.0	0.0				
WS	Treated		7.59	-8.61		-17.77	222997.3	5.3	5.2	6.6	0.9	14.1
			5.36	-8.84		-18.00	261539.0	5.4				
			6.89	-6.71		-15.87	59750.6	4.8				

Mid-shelf reef San Cristobal												
**Season**	**SAMPLE**	**GENES**	**CT (B2)**	**B2-A2**	**Average ΔCT**	**ΔΔCT**	**2^-(ΔΔCT)**	**log_{10}2^-(ΔΔCT)**	**log_{10}2^-(ΔΔCT)**	**Mean**	**STD**	**CV%**
CS	Control	COI	21.08	-0.32	-1.87	1.55	0.3	-0.5	0.0	21.1	0.58	2.77
			20.34	-1.96		-0.09	1.1	0.0				
			21.77	-3.33		-1.46	2.8	0.4				
WS	Treated		23.54	6.35		8.22	0.9	0.0	24.9	24.9	1.11	4.44
			26.25	11.11		12.98	1.1	0.0				
			25.00	11.88		13.75	1.1	0.1				
CS	Control	Green fluorescent Protein	23.89	-15.06	-16.45	1.39	0.4	-0.4	0.0	23.6	0.48	2.05
			23.90	-15.52		0.93	0.5	-0.3				
			22.87	-18.77		-2.32	5.0	0.7				
WS	Treated		6.34	-10.85		5.60	0.0	-1.7	-2.3	6.5	0.21	3.24
			6.78	-8.36		8.09	0.0	-2.4				
			6.33	-6.79		9.66	0.0	-2.9				
CS	Control	Beta tubulin	35.17	13.77	12.48	1.29	0.4	-0.4	0.0	35.4	0.51	1.43
			36.12	13.82		1.34	0.4	-0.4				
			34.95	9.85		-2.63	6.2	0.8				
WS	Treated		23.12	5.93		-6.55	93.7	2.0	23.4	23.4	0.23	0.98
			23.67	8.53		-3.95	15.5	1.2				
			23.29	10.17		-2.31	5.0	0.7				
CS	Control	Cysteine rich protein	3.45	-17.95	-19.59	34.46	0.0	-10.4	3.3	3.3	0.57	17.15
			2.59	-19.71		35.59	0.0	-10.7				
			3.98	-21.12		40.36	0.0	-12.2				
WS	Treated		32.06	14.87		11.42	0.0	-3.4	32.4	32.4	1.14	3.53
			31.14	16.00		13.70	0.0	-4.1				
			33.89	20.77		6.02	0.0	-1.8				
CS	Control	α car. anhydrase	23.64	-8.17	-9.21	5.25	0.0	-1.6	24.3	24.3	0.56	2.30
			24.32	-5.89		10.48	0.0	-3.2				
			25.01	-13.57		7.62	0.0	-2.3				
WS	Treated		13.23	-3.96		9.21	0.0	-2.8	13.7	13.7	2.02	14.74
			16.41	1.27		9.21	0.0	-2.8				
			11.53	-1.59		9.21	0.0	-2.8				
