# Supplementary material for: Seasonal Proteome Variations in Orbicella faveolata Reveal Molecular Thermal Stress Adaptations
Source: Proteomes. 2024 Jul 10;12(3):20. doi: 10.3390/proteomes12030020 (PMC11270422; doi:10.3390/proteomes12030020)
Supplement: Supplementary file 1 [file proteomes-12-00020-s001.zip › O.f. Supp. Table S3. 2.12.24.pdf]

Supplementary Table S3. Protein peptide table with BLAST (Basic Local Alignment Search Tool) of Forty-two differentially expressed proteins in *Orbicella faveolata*

Spot 1.	Heat shock protein sp H16.2	KJD18266.1	PEPTIDE SEQUENCES:		mslyhyfrpaqrs	
	Description	Max score	Total score	E value	Ident	Accession
Select seq gb|AAL29201.1|AF3:	nuclear receptor AmNR8 [Acropora millepora]	21.4	72.5	52	100%	AAL29201.1
Select seq gb|KJD18728.1|	oxaloacetate decarboxylase subunit beta [Halomonas meridiar	22.3	115	29	71%	KJD18728.1
Select seq gb|ADI50284.1|	galaxin-like 1 [Acropora millepora]	22.3	167	29	70%	ADI50284.1
Select seq gb|AAC47388.1|	Ub52 [Acropora millepora]	23.1	72.3	14	69%	AAC47388.1
Select seq gb|KJD18996.1|	2,3,4,5-tetrahydropyridine-2,6-carboxylate N-succinyltransfera	21.4	109	51	67%	KJD18996.1
Select seq dbj|BAQ19091.1|	activin 1 protein [Acropora digitifera]	21.8	139	38	65%	BAQ19091.1
Select seq gb|KJD20155.1|	glucosamine--fructose-6-phosphate aminotransferase [Halomc	21	176	71	58%	KJD20155.1
Select seq gb|KJD20155.1|	glucosamine--fructose-6-phosphate aminotransferase [Halomc	21	176	71	58%	KJD20155.1
Select seq gb|KJD17909.1|	NADH oxidase [Halomonas meridiana]	21.4	81.9	51	56%	KJD17909.1
Select seq gb|KJD18811.1|	1-deoxy-D-xylulose-5-phosphate synthase [Halomonas meridia	22.7	182	21	53%	KJD18811.1
Select seq gb|KJD18969.1|	3-octaprenyl-4-hydroxybenzoate carboxy-lyase [Halomonas me	20.6	96.5	95	53%	KJD18969.1
Select seq gb|KJD18969.1|	3-octaprenyl-4-hydroxybenzoate carboxy-lyase [Halomonas me	20.6	96.5	95	53%	KJD18969.1
Select seq gb|KJD20261.1|	amino acid transporter [Halomonas meridiana]	22.3	73.6	28	47%	KJD20261.1
Select seq gb|KJD18273.1|	deoxyguanosinetriphosphate triphosphohydrolase [Halomonas	20.6	110	94	43%	KJD18273.1
Select seq gb|KJD18273.1|	deoxyguanosinetriphosphate triphosphohydrolase [Halomonas	20.6	110	94	43%	KJD18273.1
Select seq gb|KJD18560.1|	ABC transporter substrate-binding protein [Halomonas meridia	22.3	105	28	42%	KJD18560.1
Select seq dbj|BAQ19137.1|	mothers against decapentaplegic 6/7 [Acropora digitifera]	21.4	117	52	41%	BAQ19137.1
Select seq gb|ADG44979.1|	hypothetical protein [Acropora millepora]	22.3	101	27	38%	ADG44979.1
Select seq gb|AET09723.1|	hypothetical protein C002-G4 [Acropora millepora]	22.3	116	27	38%	AET09723.1
Select seq gb|KJD18266.1|	heat-shock protein Hsp	32	47.9	0.027	33%	KJD18266.1

Spot 2.	Heat shock transcription factor	ACH53605.1	PEPTIDE SEQUENCES:		ssnihsnvpaflv	
	Description	Max Score	Total Score	E value	Per. Ident	Accession
Select seq gb|ACH53605.1|	heat shock transcription factor [Acropora millepora]	258	258	5.00E-85	100%	ACH53605.1
Select seq gb|KJD20828.1|	terminase [Halomonas meridiana]	26.9	38.2	0.46	100%	KJD20828.1
Select seq gb|KJD20631.1|	DNA gyrase subunit A [Halomonas meridiana]	22.3	154	14	100%	KJD20631.1
Select seq gb|KJD18236.1|	ubiquinone biosynthesis protein UbiB [Halomonas meridiana]	21	119	34	100%	KJD18236.1
Select seq gb|KJD18616.1|	hypothetical protein VE30_12345 [Halomonas meridiana]	19.7	56.6	78	100%	KJD18616.1
Select seq gb|KJD19941.1|	30S ribosomal protein S7 [Halomonas meridiana]	19.7	28.8	79	100%	KJD19941.1
Select seq gb|KJD18057.1|	transposase [Halomonas meridiana]	19.7	78.7	83	100%	KJD18057.1
Select seq gb|KJD18339.1|	transposase [Halomonas meridiana]	19.7	69.6	83	100%	KJD18339.1
Select seq gb|KJD19082.1|	exonuclease I [Halomonas meridiana]	19.7	136	84	100%	KJD19082.1
Select seq gb|KJD19612.1|	diguanylate cyclase [Halomonas meridiana]	19.7	173	84	100%	KJD19612.1
Select seq gb|KJD19048.1|	hypothetical protein VE30_10100 [Halomonas meridiana]	19.3	32.2	110	100%	KJD19048.1
Select seq gb|KJD18847.1|	endonuclease [Halomonas meridiana]	19.3	64.7	111	100%	KJD18847.1

Select seq gb|KJD20370.1|	DL-methionine transporter ATP-binding subunit [Halomonas m	18.9	67.5	151	100%	KJD20370.1
**Spot 3.**	**Heat shock protein 70 A1-like [Orbicella faveolata]**	**XP_020619238.1**	**PEPTIDE SEQUENCES:**		**dakrligrkfddpd**	
	Description	Max Score	Total Score	E value	Per. Ident	Accession
	heat shock protein 70 A1-like [Orbicella faveolata]	1304	1304	0	100.00%	XP_020619238.1
Select seq ref|XP_027037242.1	heat shock protein 70 A1-like [Pocillopora damicornis]	1106	1106	0	82.78%	XP_027037242.1
Select seq gb|RMX51493.1|	hypothetical protein pdam_00014847 [Pocillopora damicornis]	1106	1106	0	82.78%	RMX51493.1
Select seq gb|AKC91104.1|	heat shock protein 70 [Stylophora pistillata]	1008	1008	0	77.48%	AKC91104.1
Select seq ref|XP_022779048.1	heat shock protein 70 B2-like [Stylophora pistillata]	1006	1006	0	77.32%	XP_022779048.1
Select seq gb|AOF40010.1|	heat shock protein 70 [Stylophora pistillata]	1005	1005	0	77.94%	AOF40010.1
Select seq gb|PFX32589.1|	Heat shock protein 70 B2 [Stylophora pistillata]	1002	1002	0	78.63%	PFX32589.1
Select seq ref|XP_015748938.1	PREDICTED: heat shock 70 kDa protein 1-like isoform X2 [Acrop	1001	1001	0	74.53%	XP_015748938.1
Select seq ref|XP_027039067.1	heat shock 70 kDa protein 1-like [Pocillopora damicornis]	998	998	0	76.46%	XP_027039067.1
Select seq ref|XP_015748936.1	PREDICTED: heat shock 70 kDa protein 1-like isoform X1 [Acrop	998	998	0	75.20%	XP_015748936.1
Select seq gb|AMX23290.1|	heat shock protein 70 [Pocillopora damicornis]	998	998	0	76.31%	AMX23290.1
Select seq dbj|BAD89541.1|	heat shock protein 70 [Pocillopora damicornis]	996	996	0	76.31%	BAD89541.1
Select seq ref|XP_015774165.1	PREDICTED: galanin receptor type 2-like [Acropora digitifera]	94	94	2.00E-20	28.32%	XP_015774165.1
Select seq ref|XP_015775064.1	PREDICTED: G-protein coupled receptor 161-like [Acropora dig	90.5	90.5	2.00E-19	28.08%	XP_015775064.1
Select seq ref|XP_015774818.1	PREDICTED: probable G-protein coupled receptor No9 [Acropo	123	123	1.00E-30	28.08%	XP_015774818.1
Select seq ref|XP_015756285.1	PREDICTED: histamine H1 receptor-like isoform X1 [Acropora d	82.4	82.4	5.00E-17	27.62%	XP_015756285.1
Select seq ref|XP_015764657.1	PREDICTED: melanopsin-like [Acropora digitifera]	103	103	5.00E-24	27.54%	XP_015764657.1

**Spot 4.**	**Heat shock protein 90**	**XP_020618369.1**	**PEPTIDE SEQUENCES:**		**stkelkielipnr**	
	Description	Max Score	Total Score	E value	Per. Ident	Accession
Select seq ref|XP_020618369.1	heat shock protein HSP 90-beta-like [Orbicella faveolata]	1497	1497	0	100.00%	XP_020618369.1
Select seq ref|XP_015763591.1	PREDICTED: heat shock protein HSP 90-alpha-like [Acropora di	1203	1203	0	92.22%	XP_015763591.1
Select seq dbj|BBA20361.1|	heat shock protein 90 [Acropora tenuis]	1236	2473	0	91.84%	BBA20361.1
Select seq ref|XP_029211734.1	heat shock protein HSP 90-alpha-like [Acropora millepora]	1233	1233	0	91.70%	XP_029211734.1
Select seq ref|XP_022803600.1	heat shock protein HSP 90-beta-like [Stylophora pistillata]	1259	1259	0	91.16%	XP_022803600.1
Select seq ref|XP_027044932.1	heat shock protein HSP 90-beta-like [Pocillopora damicornis]	1252	1252	0	90.07%	XP_027044932.1
Select seq ref|XP_020896728.1	heat shock protein HSP 90-alpha [Exaiptasia pallida]	1104	1104	0	82.31%	XP_020896728.1
Select seq ref|XP_001640689.1	predicted protein [Nematostella vectensis]	1144	1144	0	81.25%	XP_001640689.1
Select seq ref|XP_028677486.1	heat shock protein HSP 90-alpha [Erpetoichthys calabaricus]	1092	1092	0	80.03%	XP_028677486.1
Select seq gb|EEC18473.1|	Hsp90 protein, putative [Ixodes scapularis]	1123	1123	0	80.03%	EEC18473.1
Select seq ref|XP_029834818.1	heat shock protein HSP 90-alpha [Ixodes scapularis]	1122	1122	0	80.03%	XP_029834818.1

Appendix A.3 (continued)

Spot 5. **Endoplasmin-like [Orbicella faveolata]** **XP_020606735.1** **PEPTIDE SEQUENCES:** **aqmkelreka**

	Description	Max Score	Total Score	E value	Per. Ident	Accession
Select seq ref|XP_020606735.1	endoplasmin-like [Orbicella faveolata]	514	514	0	100.00%	XP_020606735.1
Select seq ref|XP_020606732.1	endoplasmin-like isoform X1 [Orbicella faveolata]	514	514	2.00E-175	100.00%	XP_020606732.1
Select seq gb|RMX38721.1|	hypothetical protein pdam_00012456 [Pocillopora damicornis]	442	885	4.00E-147	95.28%	RMX38721.1
Select seq ref|XP_027052700.1	endoplasmin-like [Pocillopora damicornis]	440	440	1.00E-146	95.26%	XP_027052700.1
Select seq ref|XP_022802311.1	endoplasmin-like [Stylophora pistillata]	438	438	1.00E-145	94.86%	XP_022802311.1
Select seq ref|XP_029205156.1	endoplasmin-like [Acropora millepora]	431	431	6.00E-143	88.14%	XP_029205156.1
Select seq ref|XP_020907283.1	endoplasmin [Exaiptasia pallida]	430	430	2.00E-142	86.22%	XP_020907283.1
Select seq ref|XP_001637407.1	predicted protein [Nematostella vectensis]	412	412	7.00E-136	81.82%	XP_001637407.1
Select seq ref|XP_028407863.1	LOW QUALITY PROTEIN: endoplasmin-like [Dendronephthya gi	409	409	2.00E-134	79.38%	XP_028407863.1
Select seq gb|KFM57735.1|	Endoplasmin [Stegodyphus mimosarum]	389	389	2.00E-127	76.10%	KFM57735.1

Spot 6. **Cytochrome b [*Montastraea faveolata*]** **Q762T3** **PEPTIDE SEQUENCES:** **gytvdflpypvse**

	Description	Total Score	E value	Per. Ident	Accession	
Select seq dbj|BAD11494.0|	cytochrome b [Orbicella annularis]	748	100%	0	100.00%	YP_271926.1
Select seq dbj|BAD11494.1|	cytochrome b [Echinopora pacificus]	746	100%	0	99.21%	BAD11494.1
Select seq dbj|BAD11495.1|	cytochrome b [Echinopora pacificus]	746	100%	0	98.94%	BAD11495.1
Select seq dbj|BAD11488.1|	cytochrome b [Cyphastrea serailia]	744	100%	0	98.68%	BAD11488.1
Select seq gb|AEL17177.1|	cytochrome b [Favia pallida]	743	100%	0	99.21%	AEL17177.1
Select seq dbj|BAD11490.1|	cytochrome b [Cyphastrea chalcidicum]	743	100%	0	98.15%	BAD11490.1
Select seq ref|YP_007317708.1	cytochrome b [Platygyra carnosa]	742	100%	0	98.94%	YP_007317708.1
Select seq dbj|BAD11497.1|	cytochrome b [Favia stelligera]	742	100%	0	99.21%	BAD11497.1
Select seq ref|YP_009378909.1	cytochrome b [Favites pentagona]	741	100%	0	98.68%	YP_009378909.1
Select seq dbj|BAD11501.1|	cytochrome b [Favites halicora]	741	100%	0	98.68%	BAD11501.1

Spot 7. **Cytochrome oxidase subunit I [Montastraea faveolata]** **AHA90940.1** **PEPTIDE SEQUENCES:** **sfwllppalflll**

	Description	Total Score	Query	E value	Per. Ident	Accession
Select seq gb|AHA90940.1|	cytochrome oxidase subunit I [Orbicella faveolata]	245	100%	7.00E-82	100.00%	AHA90940.1
Select seq gb|AHA90942.1|	cytochrome oxidase subunit I [Orbicella faveolata]	245	100%	5.00E-81	99.20%	AHA90942.1
Select seq gb|AHA90943.1|	cytochrome oxidase subunit I [Orbicella faveolata]	243	100%	6.00E-81	99.20%	AHA90943.1
Select seq gb|AHA90945.1|	cytochrome oxidase subunit I [Orbicella faveolata]	244	100%	6.00E-81	99.20%	AHA90945.1
Select seq gb|AHA90941.1|	cytochrome oxidase subunit I [Orbicella faveolata]	244	100%	8.00E-81	99.20%	AHA90941.1
Select seq gb|AFP73387.1|	cytochrome oxidase subunit I [Pseudodiploria strigosa]	243	100%	4.00E-80	97.60%	AFP73387.1
Select seq gb|AAS17027.1|	cytochrome oxidase subunit I [Orbicella annularis]	244	100%	4.00E-80	99.20%	AAS17027.1
Select seq gb|ACJ09533.1|	cytochrome oxidase subunit I [Platygyra pini]	242	100%	6.00E-80	98.40%	ACJ09533.1

Select seq gb|ABY85346.1|	cytochrome oxidase subunit I [Favites cf. valenciennesi DH-201	243	100%	6.00E-80	98.40%	ABY85346.1

Appendix A.3 (continued)

Select seq gb|ABY85297.1|	cytochrome oxidase subunit I [Favia danae]	486	100%	9.00E-80	98.40%	ABY85297.1
Select seq dbj|BAD11414.1|	cytochrome oxidase I [Orbicella annularis]	243	100%	9.00E-80	99.20%	BAD11414.1

**Spot 8.**	**Cytochrome P450 74A**	**ACD42778.1**	**PEPTIDE SEQUENCES:**		**sdvieilklavna**	
	Description	Total Score	Query	E value	Per. Ident	Accession
Select seq gb|ACD42778.1|	cytochrome P450 74A [Acropora palmata]	904	904	0	100.00%	ACD42778.1
Select seq ref|XP_015765607.1	PREDICTED: allene oxide synthase, chloroplastic-like isoform X1	872	872	0	96.06%	XP_015765607.1
Select seq ref|XP_029184931.1	allene oxide synthase, chloroplastic-like isoform X1 [Acropora r	867	867	0	96.06%	XP_029184931.1
Select seq ref|XP_015765609.1	PREDICTED: allene oxide synthase, chloroplastic-like isoform X2	855	855	0	94.90%	XP_015765609.1
Select seq ref|XP_029184932.1	allene oxide synthase, chloroplastic-like isoform X2 [Acropora r	850	850	0	94.90%	XP_029184932.1
Select seq ref|XP_020613338.1	allene oxide synthase-like [Orbicella faveolata]	644	644	0	67.44%	XP_020613338.1
Select seq ref|XP_027060358.1	allene oxide synthase-like isoform X1 [Pocillopora damicornis]	634	634	0	66.90%	XP_027060358.1
Select seq ref|XP_027060359.1	allene oxide synthase-like isoform X2 [Pocillopora damicornis]	632	632	0	66.90%	XP_027060359.1
Select seq ref|XP_022797645.1	allene oxide synthase-like isoform X2 [Stylophora pistillata]	622	622	0	65.59%	XP_022797645.1
Select seq gb|PFX21200.1|	Allene oxide synthase [Stylophora pistillata]	619	873	0	65.59%	PFX21200.1

**Spot 9.**	**Activin 1**	**BAQ19091.1**	**PEPTIDE SEQUENCES:**		**rcpscndsakene**	
	Description	Max Score	Total Score	E value	Per. Ident	Accession
Select seq ref|XP_029198106.0	PREDICTED: growth/differentiation factor 8-like [Acropora digit	885	885	100%	100.00%	XP_015761587.1
Select seq ref|XP_029198106.1	growth/differentiation factor 8-like [Acropora millepora]	861	861	0	97.17%	XP_029198106.1
Select seq ref|XP_015761586.1	PREDICTED: growth/differentiation factor 8-like [Acropora digit	478	478	4.00E-164	57.64%	XP_015761586.1
Select seq ref|XP_029198105.1	growth/differentiation factor 8-like [Acropora millepora]	476	476	2.00E-163	57.64%	XP_029198105.1
Select seq ref|XP_020629122.1	inhibin beta B chain-like isoform X3 [Orbicella faveolata]	286	286	7.00E-89	42.08%	XP_020629122.1
Select seq ref|XP_020629118.1	inhibin beta B chain-like isoform X2 [Orbicella faveolata]	286	286	8.00E-89	42.08%	XP_020629118.1
Select seq ref|XP_020629111.1	inhibin beta B chain-like isoform X1 [Orbicella faveolata]	286	286	1.00E-88	41.86%	XP_020629111.1
Select seq ref|XP_027045428.1	inhibin beta B chain-like [Pocillopora damicornis]	263	263	9.00E-80	37.50%	XP_027045428.1
Select seq ref|XP_022785216.1	inhibin beta B chain-like [Stylophora pistillata]	258	258	9.00E-78	37.18%	XP_022785216.1
Select seq ref|XP_019632507.1	PREDICTED: growth/differentiation factor 8-like [Branchiostom	147	147	5.00E-36	30.59%	XP_019632507.1
Select seq gb|ABF61781.1|	activin [Nematostella vectensis]	144	144	5.00E-35	27.11%	ABF61781.1

**Spot 10.**	**NADH-ubiquinone oxidoreductase chain 1**	**Q4G6D2**	**PEPTIDE SEQUENCES:**		**aqmkelreka**	
	Description	Max Score	Total Score	E value	Per. Ident	Accession
Select seq gb|ATC69366.1|	NADH dehydrogenase subunit 1 [Acropora pruinosa]	468	468	8.00E-168	74.69%	ATC69366.1
Select seq ref|YP_008815375.1	NADH dehydrogenase subunit 1 [Acropora humilis]	469	469	9.00E-168	74.69%	YP_008815375.1
Select seq gb|ATC69390.1|	NADH dehydrogenase subunit 1 [Acropora nana]	468	468	9.00E-168	74.69%	ATC69390.1
Select seq gb|ABD17707.1|	NADH dehydrogenase subunit 1 [Acropora acuminata]	70.5	70.5	8.00E-16	74.47%	ABD17707.1
Select seq ref|NP_612817.1|	NADH dehydrogenase subunit 1 [Acropora tenuis]	467	467	6.00E-167	74.38%	NP_612817.1

Select seq gb|ABD17695.1|	NADH dehydrogenase subunit 1 [Acropora acuminata]	211	211	5.00E-69	72.86%	ABD17695.1

Appendix A.3 (continued)

**Spot 11.**	**Thioredoxin reductase [A. millepora**	**AFI99106.2**	**PEPTIDE SEQUENCES:**		**spigttwglggt**	
	Description	Total Score	Query	E value	Per. Ident	Accession
Select seq gb|AFI99106.2|	thioredoxin reductase [Acropora millepora]	1227	1227	0	100.00%	AFI99106.2
Select seq ref|XP_029185498.1	thioredoxin reductase 1, cytoplasmic-like isoform X3 [Acropora	1221	1221	0	99.83%	XP_029185498.1
Select seq ref|XP_029185497.1	thioredoxin reductase 1, cytoplasmic-like isoform X2 [Acropora	1221	1221	0	99.83%	XP_029185497.1
Select seq ref|XP_029185496.1	thioredoxin reductase 1, cytoplasmic-like isoform X1 [Acropora	1220	1220	0	99.83%	XP_029185496.1
Select seq ref|XP_029185499.1	thioredoxin reductase 1, cytoplasmic-like isoform X4 [Acropora	1155	1155	0	99.82%	XP_029185499.1
Select seq ref|XP_020605984.1	thioredoxin reductase 1, cytoplasmic-like isoform X1 [Orbicella	1053	1053	0	83.25%	XP_020605984.1
Select seq ref|XP_020605985.1	thioredoxin reductase 1, cytoplasmic-like isoform X2 [Orbicella	1051	1051	0	83.25%	XP_020605985.1
Select seq ref|XP_001630291.1	predicted protein [Nematostella vectensis]	895	895	0	70.71%	XP_001630291.1
Select seq ref|XP_015763837.1	PREDICTED: thioredoxin reductase 3-like isoform X2 [Acropora	790	790	0	99.21%	XP_015763837.1
Select seq ref|XP_015763830.1	PREDICTED: thioredoxin reductase 3-like isoform X1 [Acropora	790	790	0	99.21%	XP_015763830.1
Select seq gb|RXM93140.1|	Thioredoxin reductase 3 [Acipenser ruthenus]	783	783	0	65.31%	RXM93140.1

**Spot 12.**	**Apextrin**	**ADT3F5**	**PEPTIDE SEQUENCES:**		**mfvilgvvll**	
	Description	Total Score	Query	E value	Per. Ident	Accession
Select seq gb|ABK63971.2|	apextrin [Acropora millepora]	35.4	35.4	2.00E-06	100%	ABK63971.2
Select seq gb|KJD18891.1|	sulfite oxidase [Halomonas meridiana]	24	24	0.011	78%	KJD18891.1
Select seq gb|KJD20797.1|	ABC transporter permease [Halomonas meridiana]	21	21	0.095	100%	KJD20797.1
Select seq gb|KJD18783.1|	cytochrome B [Halomonas meridiana]	21	45.6	0.096	70%	KJD18783.1
Select seq gb|KJD19402.1|	C4-dicarboxylate ABC transporter permease [Halomonas meric	21	21	0.096	100%	KJD19402.1
Select seq gb|KJD18161.1|	hypothetical protein VE30_14675 [Halomonas meridiana]	21	49	0.096	100%	KJD18161.1
Select seq gb|ABY74499.1|	integrin beta 2 [Acropora millepora]	21	21	0.096	100%	ABY74499.1
Select seq gb|KJD18722.1|	cytochrome C biogenesis protein CcmC [Halomonas meridiana	20.6	20.6	0.13	70%	KJD18722.1
Select seq gb|KJD19494.1|	cell division protein FtsQ [Halomonas meridiana]	20.6	20.6	0.13	100%	KJD19494.1
Select seq gb|KJD19467.1|	NADH dehydrogenase [Halomonas meridiana]	20.6	38.2	0.13	100%	KJD19467.1

**Spot 13.**	**ATP synthase**	**YP_271955**	**PEPTIDE SEQUENCES:**		**fflvflnifglc**	
	Description	Total Score	Query	E value	Per. Ident	Accession
Select seq ref|YP_009428246.0	ATP synthase F0 subunit 6 [Orbicella annularis]	436	100%	100.00%	YP_271929.1	
Select seq ref|YP_009428246.1	ATP synthase F0 subunit 6 [Favites abdita]	431	3.00E-152	99.11%	YP_009428246.1	
Select seq ref|YP_009679161.1	ATP synthase subunit 6 [Dipsastraea rotumana]	430	6.00E-152	98.67%	YP_009679161.1	
Select seq ref|YP_009654478.1	ATP synthase F0 subunit 6 [Hydnophora exesa]	859	1.00E-151	98.22%	YP_009654478.1	
Select seq ref|YP_654359.1|	ATP synthase F0 subunit 6 [Mussa angulosa]	429	3.00E-151	97.78%	YP_654359.1	

Select seq ref|YP_007317711.1	ATP synthase F0 subunit 6 [Platygyra carnosa]	427	7.00E-151	97.78%	YP_007317711.1
Select seq ref|YP_009547093.1	ATP synthase F0 subunit 6 [Echinophyllia aspera]	427	1.00E-150	96.89%	YP_009547093.1

Appendix A.3 (continued)

Select seq ref|YP_009378912.1	ATP synthase F0 subunit 6 [Favites pentagona]	427	1.00E-150	97.33%	YP_009378912.1
Select seq ref|YP_654346.1|	ATP synthase F0 subunit 6 [Colpophyllia natans]	427	1.00E-150	97.78%	YP_654346.1

Spot 14. **ATP synthase subunit 8** **Q8SJB2** **PEPTIDE SEQUENCES:** **tqyrwtllvl**

	Description	Total Score	Query Cover	E value	Per. Ident	Accession
Select seq ref|NP_612827.1|	ATP synthase F0 subunit 8 [Acropora tenuis]	146	146	5.00E-48	100.00%	NP_612827.1
Select seq ref|XP_015780370.1	PREDICTED: adenosine receptor A3-like [Acropora digitifera]	25.4	25.4	3.8	50.00%	XP_015780370.1
Select seq ref|XP_015768528.1	PREDICTED: fibronectin-like [Acropora digitifera]	24.6	24.6	7.5	44.00%	XP_015768528.1

Spot 15. **ATPase subunit 6, (ATP synthase subunit 6)** **Q9TBW1** **PEPTIDE SEQUENCES:** **fmpsgaplgl**

	Description	Total Score	Query	E value	Per. Ident	Accession
Select seq ref|NP_612821.1|	ATP synthase F0 subunit 6 [Acropora tenuis]	448	896	1.00E-158	100.00%	NP_612821.1
Select seq ref|YP_009029625.1	ATP synthase F0 subunit 6 [Isopora palifera]	446	446	6.00E-158	99.57%	YP_009029625.1
Select seq ref|YP_009472225.1	ATP synthase F0 subunit 6 [Montipora aequituberculata]	444	444	4.00E-157	98.71%	YP_009472225.1
Select seq ref|YP_214919.1|	ATP synthase F0 subunit 6 [Anacropora matthai]	442	442	3.00E-156	98.28%	YP_214919.1
Select seq gb|AHY04480.1|	ATP synthase F0 subunit 6 [Alveopora sp. MFL-2014]	439	439	5.00E-155	98.28%	AHY04480.1
Select seq ref|YP_009029612.1	ATP synthase F0 subunit 6 [Astreopora explanata]	437	437	3.00E-154	97.41%	YP_009029612.1
Select seq ref|YP_009545481.1	ATP synthase F0 subunit 6 [Alveopora japonica]	431	431	3.00E-152	96.98%	YP_009545481.1
Select seq ref|YP_004581257.1	ATP synthase F0 subunit 6 [Fungiacyathus stephanus]	415	830	1.00E-145	90.95%	YP_004581257.1
Select seq ref|YP_009159301.1	ATP synthase F0 subunit 6 [Dendrophyllia arbuscula]	414	414	2.00E-145	91.38%	YP_009159301.1
Select seq ref|YP_009122416.1	ATP synthase F0 subunit 6 [Pseudosiderastrea tayami]	413	413	5.00E-145	94.83%	YP_009122416.1
Select seq dbj|BAA85613.1|	ATPase 6 [Isopora brueggemanni]	412	412	8.00E-145	100.00%	BAA85613.1

Spot 16. **Toll-like receptor 2** **XP_020617122** **PEPTIDE SEQUENCES:** **qwcnfelqqaiyqhl**

	Description	Total Score	Query	E value	Per. Ident	Accession
Select seq ref|XP_020617122.1	toll-like receptor 2 isoform X4 [Orbicella faveolata]	438	100%	1.00E-155	100.00%	XP_020617122.1
Select seq ref|XP_020617118.1	toll-like receptor 2 isoform X1 [Orbicella faveolata]	431	100%	3.00E-152	94.64%	XP_020617118.1
Select seq ref|XP_020617121.1	toll-like receptor 2 isoform X3 [Orbicella faveolata]	429	100%	9.00E-152	94.62%	XP_020617121.1
Select seq ref|XP_020630889.1	uncharacterized protein LOC110067885 isoform X3 [Orbicella f	187	84%	6.00E-54	53.01%	XP_020630889.1
Select seq ref|XP_020630888.1	uncharacterized protein LOC110067885 isoform X2 [Orbicella f	187	84%	9.00E-54	53.01%	XP_020630888.1
Select seq ref|XP_020630887.1	uncharacterized protein LOC110067885 isoform X1 [Orbicella f	187	84%	2.00E-53	53.01%	XP_020630887.1
Select seq ref|XP_015769651.1	PREDICTED: toll-like receptor 4 isoform X2 [Acropora digitifera	157	91%	3.00E-43	41.62%	XP_015769651.1
Select seq ref|XP_015769650.1	PREDICTED: uncharacterized protein LOC107348148 isoform X	157	84%	4.00E-43	42.78%	XP_015769650.1

Select seq ref|XP_027036966.1	uncharacterized protein LOC113665468 [Pocillopora damicorn	155	93%	3.00E-41	39.90%	XP_027036966.1
Select seq gb|RMX51749.1|	hypothetical protein pdam_00011740 [Pocillopora damicornis]	249	93%	2.00E-39	41.21%	RMX51749.1

Appendix A.3 (continued)

**Spot 17.**	**Mini-collagen**	**AAM74869.1**	**PEPTIDE SEQUENCES:**		**ekreaspcgyg**	
	Description	Total Score	Query	E value	Per. Ident	Accession
Select seq gb|AAM74852.1|	mini-collagen [Acropora cervicornis]	94	94	100%	5.00E-24	AAM74852.1
Select seq gb|AAM74869.1|	mini-collagen [Acropora palmata]	93.6	93.6	100%	7.00E-24	AAM74869.1
Select seq gb|AAM74878.1|	mini-collagen [Acropora palmata]	91.3	91.3	100%	5.00E-23	AAM74878.1
Select seq ref|XP_015771910.1	PREDICTED: putative cuticle collagen 155 [Acropora digitifera]	94.4	94.4	100%	9.00E-23	XP_015771910.1
Select seq dbj|BAA06407.1|	mini-collagen [Acropora donei]	92.8	92.8	100%	3.00E-22	BAA06407.1
Select seq ref|XP_029208364.1	basic proline-rich protein-like [Acropora millepora]	92.8	92.8	100%	3.00E-22	XP_029208364.1
Select seq ref|XP_022804287.1	cuticle collagen 34-like [Stylophora pistillata]	53.1	53.1	60%	6.00E-07	XP_022804287.1
Select seq ref|XP_028415759.1	cuticle collagen 1-like [Dendronephthya gigantea]	40.8	40.8	48%	0.017	XP_028415759.1
Select seq gb|AAM74851.1|	mini-collagen [Acropora nasuta]	38.9	38.9	60%	0.032	AAM74851.1
Select seq ref|XP_001641074.1	predicted protein [Nematostella vectensis]	39.7	39.7	53%	0.04	XP_001641074.1
Select seq ref|XP_020625137.1	cuticle collagen 14-like [Orbicella faveolata]	40	40	55%	0.066	XP_020625137.1

Appendix A.3 (continued)

**Spot 18.**	**2-acylglycerol O-acyltransferase 1-like [Orbicella faveolata]**	**XP_020602679.1**	**PEPTIDE SEQUENCES:**		**grrsnfvrrlrv**	
	Description	Total Score	Query	E value	Per. Ident	Accession
Select seq ref|XP_020602679.1	2-acylglycerol O-acyltransferase 1-like [Orbicella faveolata]	688	100%	0	100.00%	XP_020602679.1
Select seq ref|XP_029187260.1	2-acylglycerol O-acyltransferase 1-like [Acropora millepora]	1264	99%	0	89.29%	XP_029187260.1
Select seq ref|XP_022791455.1	2-acylglycerol O-acyltransferase 1-like [Stylophora pistillata]	625	100%	0	88.43%	XP_022791455.1
Select seq ref|XP_027048695.1	2-acylglycerol O-acyltransferase 1-like [Pocillopora damicornis]	619	99%	0	88.10%	XP_027048695.1
Select seq ref|XP_015752089.1	PREDICTED: 2-acylglycerol O-acyltransferase 1-like [Acropora d	611	99%	0	88.69%	XP_015752089.1
Select seq ref|XP_028401261.1	2-acylglycerol O-acyltransferase 2-A-like [Dendronephthya giga	541	99%	0	75.45%	XP_028401261.1
Select seq ref|XP_001630435.1	predicted protein [Nematostella vectensis]	541	99%	0	76.79%	XP_001630435.1
Select seq ref|XP_020902957.1	2-acylglycerol O-acyltransferase 1 [Exaiptasia pallida]	535	99%	0	73.51%	XP_020902957.1
Select seq ref|XP_008591845.1	PREDICTED: 2-acylglycerol O-acyltransferase 1 [Galeopterus va	392	98%	2.00E-133	56.33%	XP_008591845.1
Select seq ref|XP_017390802.1	PREDICTED: 2-acylglycerol O-acyltransferase 1 isoform X1 [Ceb	391	98%	6.00E-133	56.93%	XP_017390802.1
Select seq ref|XP_027046098.1	2-acylglycerol O-acyltransferase 2-like [Pocillopora damicornis]	391	99%	6.00E-133	55.22%	XP_027046098.1
Select seq ref|XP_002749878.1	PREDICTED: 2-acylglycerol O-acyltransferase 1 [Callithrix jacchu	391	98%	9.00E-133	56.93%	XP_002749878.1
Select seq ref|XP_003925530.1	PREDICTED: 2-acylglycerol O-acyltransferase 1 [Saimiri bolivien	390	98%	2.00E-132	56.63%	XP_003925530.1

Spot 19.	Integrin beta	XP_020602679.1	PEPTIDE SEQUENCES:		grrsnfvrrlrv	
	Description	Total Score	Query	E value	Per. Ident	Accession
Select seq gb|AAB66910.1|	integrin subunit betaCn1 [Acropora millepora]	1630	1630	0	100.00%	AAB66910.1
Select seq ref|XP_015755972.1	PREDICTED: integrin beta-1-like [Acropora digitifera]	1094	1094	0	73.96%	XP_015755972.1
Select seq ref|XP_015762689.1	PREDICTED: uncharacterized protein LOC107341737 isoform X	30	30	6.1	66.67%	XP_015762689.1

Appendix A.3 (continued)

Select seq ref|XP_015762690.1	PREDICTED: uncharacterized protein LOC107341738 isoform X	30	30	6.3	66.67%	XP_015762690.1
Select seq ref|XP_015762686.1	PREDICTED: uncharacterized protein LOC107341736 [Acropora	30	30	6.9	66.67%	XP_015762686.1
Select seq ref|XP_015765150.1	PREDICTED: versican core protein-like isoform X1 [Acropora di	30	30	6.7	51.52%	XP_015765150.1
Select seq ref|XP_015775330.1	PREDICTED: uncharacterized protein LOC107353528 [Acropora	29.6	29.6	6.4	50.00%	XP_015775330.1
Select seq ref|XP_015755165.1	PREDICTED: ALK tyrosine kinase receptor-like [Acropora digitife	29.6	29.6	9.8	46.67%	XP_015755165.1
Select seq ref|XP_015759479.1	PREDICTED: attractin-like protein 1 [Acropora digitifera]	32.7	32.7	1	46.15%	XP_015759479.1
Select seq gb|ARA71555.1|	MAM and LDL-receptor domain-containing protein [Acropora	32.3	32.3	2	41.94%	ARA71555.1

Spot 20.	Actin	ABY40470.1	PEPTIDE SEQUENCES:		mkcdvdirkdlyant	
	Description	Max Score	Total Score	E value	Per. Ident	Accession
Select seq gb|ABY40470.1|	actin [Acropora millepora]	323	323	1.00E-115	100.00%	ABY40470.1
Select seq ref|XP_015762563.1	PREDICTED: actin, cytoplasmic-like [Acropora digitifera]	324	324	1.00E-112	100.00%	XP_015762563.1
Select seq ref|XP_015751952.1	PREDICTED: actin-15B [Acropora digitifera]	309	309	1.00E-106	94.19%	XP_015751952.1
Select seq ref|XP_015753791.1	PREDICTED: actin, cytoplasmic [Acropora digitifera]	306	306	8.00E-106	93.55%	XP_015753791.1
Select seq ref|XP_015753792.1	PREDICTED: actin-3 [Acropora digitifera]	301	301	8.00E-104	92.26%	XP_015753792.1
Select seq ref|XP_015768232.1	PREDICTED: uncharacterized protein LOC107346890 [Acropora	306	497	5.00E-101	92.90%	XP_015768232.1
Select seq gb|AEX31637.1|	beta-actin [Acropora pruinosa]	157	157	6.00E-51	96.05%	AEX31637.1
Select seq ref|XP_015748011.1	PREDICTED: alpha-centractin [Acropora digitifera]	162	162	3.00E-50	49.68%	XP_015748011.1
Select seq ref|XP_015764694.1	PREDICTED: LOW QUALITY PROTEIN: actin-related protein 2-lik	139	139	2.00E-40	41.77%	XP_015764694.1
Select seq ref|XP_015763253.1	PREDICTED: actin-like [Acropora digitifera]	114	114	1.00E-31	39.10%	XP_015763253.1
Select seq ref|XP_015768231.1	PREDICTED: actin, alpha skeletal muscle-like [Acropora digitife	102	102	6.00E-29	52.81%	XP_015768231.1
Select seq ref|XP_015757596.1	PREDICTED: actin-like protein 6A [Acropora digitifera]	108	108	1.00E-28	40.80%	XP_015757596.1
Select seq ref|XP_015762522.1	PREDICTED: actin [Acropora digitifera]	100	100	2.00E-26	72.86%	XP_015762522.1
Select seq ref|XP_015762037.1	PREDICTED: actin-related protein 6-like [Acropora digitifera]	80.5	80.5	2.00E-19	27.54%	XP_015762037.1
Select seq ref|XP_015748134.1	PREDICTED: actin-related protein 6-like [Acropora digitifera]	79	79	7.00E-18	26.44%	XP_015748134.1
Select seq ref|XP_015765936.1	PREDICTED: actin-related protein 5-like [Acropora digitifera]	65.1	65.1	6.00E-13	30.21%	XP_015765936.1
Select seq ref|XP_015765935.1	PREDICTED: actin-related protein 5-like [Acropora digitifera]	62	62	7.00E-12	36.00%	XP_015765935.1
Select seq ref|XP_015757125.1	PREDICTED: actin-related protein 3-like [Acropora digitifera]	30.8	30.8	0.33	31.91%	XP_015757125.1
Select seq ref|XP_015779499.1	PREDICTED: dynein heavy chain domain-containing protein 1-li	27.3	27.3	6.2	36.59%	XP_015779499.1

Spot 21. **Beta-tubulin** **AHZ61618.1** **PEPTIDE SEQUENCES:** **natlsvhqlventdet**

	Description	Total Score	Query	Per. Ident	Accession	
Select seq gb|AHZ61578.1|	beta-tubulin [Acropora cervicornis]	163	163	1.00E-54	100.00%	AHZ61578.1
Select seq ref|XP_015747460.1	PREDICTED: tubulin beta-4B chain isoform X4 [Acropora digitife	169	169	2.00E-52	100.00%	XP_015747460.1
Select seq ref|XP_015747441.1	PREDICTED: tubulin beta-4B chain isoform X1 [Acropora digitife	169	169	2.00E-52	100.00%	XP_015747441.1
Select seq ref|XP_015761439.1	PREDICTED: tubulin beta chain-like [Acropora digitifera]	161	161	5.00E-50	94.94%	XP_015761439.1
Select seq ref|XP_015768684.1	PREDICTED: tubulin alpha-1 chain [Acropora digitifera]	75.1	75.1	1.00E-17	42.86%	XP_015768684.1

Appendix A.3 (continued)

Select seq ref|XP_015773084.1	PREDICTED: tubulin alpha-3 chain [Acropora digitifera]	72.8	72.8	5.00E-17	38.96%	XP_015773084.1
Select seq ref|XP_015771897.1	PREDICTED: tubulin alpha chain-like [Acropora digitifera]	73.2	73.2	6.00E-17	40.26%	XP_015771897.1
Select seq ref|XP_015747471.1	PREDICTED: LOW QUALITY PROTEIN: tubulin alpha-8 chain-like	72.4	72.4	1.00E-16	38.96%	XP_015747471.1
Select seq ref|XP_015770470.1	PREDICTED: tubulin gamma-1 chain isoform X1 [Acropora digit	60.1	60.1	3.00E-12	37.18%	XP_015770470.1
Select seq ref|XP_015770471.1	PREDICTED: tubulin gamma-1 chain isoform X2 [Acropora digit	60.1	60.1	3.00E-12	37.18%	XP_015770471.1
Select seq ref|XP_015757049.1	PREDICTED: tubulin beta chain-like [Acropora digitifera]	50.8	50.8	6.00E-09	57.14%	XP_015757049.1
Select seq ref|XP_015757384.1	PREDICTED: tubulin epsilon chain-like [Acropora digitifera]	45.4	45.4	5.00E-07	38.81%	XP_015757384.1

Spot 22. **Gelsolin** **XP_012559373.1** **PEPTIDE SEQUENCES:** **eeelkydvhfwige**

	Description	Max Score	Total Score	E value	Per. Ident	Accession
Select seq ref|XP_015766322.1	PREDICTED: gelsolin-like protein 1 [Acropora digitifera]	294	294	2.00E-99	60.34%	XP_015766322.1
Select seq ref|XP_015766340.1	PREDICTED: gelsolin-like protein 1 [Acropora digitifera]	153	153	1.00E-45	51.95%	XP_015766340.1
Select seq ref|XP_015766323.1	PREDICTED: gelsolin-like protein 1 [Acropora digitifera]	350	350	9.00E-120	49.86%	XP_015766323.1
Select seq ref|XP_015765697.1	PREDICTED: advillin-like [Acropora digitifera]	204	318	5.00E-60	40.00%	XP_015765697.1
Select seq ref|XP_015766301.1	PREDICTED: gelsolin-like protein 1 [Acropora digitifera]	127	127	1.00E-34	33.74%	XP_015766301.1
Select seq ref|XP_015752858.1	PREDICTED: protein flightless-1 homolog [Acropora digitifera]	103	103	4.00E-24	26.16%	XP_015752858.1
Select seq ref|XP_015776614.1	PREDICTED: supervillin-like [Acropora digitifera]	33.5	33.5	0.29	24.72%	XP_015776614.1
Select seq ref|XP_015750047.1	PREDICTED: supervillin-like [Acropora digitifera]	35	35	0.08	20.51%	XP_015750047.1

Spot 23. **Myosin** **XP_001637809.1** **PEPTIDE SEQUENCES:** **sgagktestkfiicq**

	Description	Max Score	Total Score	E value	Per. Ident	Accession
Select seq ref|XP_015769762.1	PREDICTED: unconventional myosin-VIIa-like [Acropora digitife	758	758	0	68.12%	XP_015769762.1
Select seq ref|XP_015769761.1	PREDICTED: myosin-1-like [Acropora digitifera]	77.8	77.8	2.00E-17	50.00%	XP_015769761.1
Select seq ref|XP_015779248.1	PREDICTED: LOW QUALITY PROTEIN: unconventional myosin-X	164	278	8.00E-43	48.50%	XP_015779248.1
Select seq ref|XP_015756084.1	PREDICTED: unconventional myosin-VI-like [Acropora digitifera	174	283	4.00E-46	45.45%	XP_015756084.1
Select seq ref|XP_015761879.1	PREDICTED: myosin-VIIa-like [Acropora digitifera]	236	347	4.00E-67	44.04%	XP_015761879.1
Select seq ref|XP_015766505.1	PREDICTED: unconventional myosin-XV-like [Acropora digitifer	407	407	4.00E-126	42.34%	XP_015766505.1
Select seq ref|XP_015749129.1	PREDICTED: unconventional myosin-VIIa-like [Acropora digitife	200	200	2.00E-60	41.18%	XP_015749129.1
Select seq ref|XP_015752126.1	PREDICTED: unconventional myosin-Ib-like [Acropora digitifera	352	352	5.00E-109	39.46%	XP_015752126.1
Select seq ref|XP_015758662.1	PREDICTED: unconventional myosin-Ie-like isoform X2 [Acropo	340	340	4.00E-105	39.25%	XP_015758662.1

Select seq ref|XP_015758661.1	PREDICTED: unconventional myosin-Ie-like isoform X1 [Acropo	339	339	9.00E-103	39.18%	XP_015758661.1
Select seq ref|XP_015758734.1	PREDICTED: myosin-IB-like [Acropora digitifera]	340	340	5.00E-109	38.31%	XP_015758734.1
Select seq ref|XP_015759619.1	PREDICTED: unconventional myosin-VIIa-like [Acropora digitife	358	358	7.00E-113	38.17%	XP_015759619.1
Select seq ref|XP_015779047.1	PREDICTED: unconventional myosin-Id-like [Acropora digitifera	326	326	1.00E-100	37.50%	XP_015779047.1
Select seq ref|XP_015768410.1	PREDICTED: unconventional myosin-Vb-like [Acropora digitifera	321	321	2.00E-96	37.08%	XP_015768410.1
Select seq ref|XP_015774550.1	PREDICTED: myosin heavy chain, striated muscle-like [Acropora	327	327	3.00E-98	36.84%	XP_015774550.1
Select seq ref|XP_015752855.1	PREDICTED: myosin-10-like [Acropora digitifera]	321	321	2.00E-96	36.63%	XP_015752855.1
Select seq ref|XP_015760291.1	PREDICTED: unconventional myosin-XVIIIa-like [Acropora digiti	90.1	131	1.00E-18	36.42%	XP_015760291.1
Select seq ref|XP_015778219.1	PREDICTED: myosin-9-like [Acropora digitifera]	187	187	4.00E-51	36.36%	XP_015778219.1

Appendix A.3 (continued)

Select seq ref|XP_015757748.1	PREDICTED: unconventional myosin-XIX-like [Acropora digitifer	297	297	4.00E-90	35.70%	XP_015757748.1
Select seq ref|XP_015761283.1	PREDICTED: myosin-IIIa-like [Acropora digitifera]	233	297	2.00E-66	35.68%	XP_015761283.1
Select seq ref|XP_015769070.1	PREDICTED: unconventional myosin-If-like [Acropora digitifera]	293	293	4.00E-90	35.61%	XP_015769070.1
Select seq ref|XP_015749603.1	PREDICTED: unconventional myosin-Ie-like [Acropora digitifera	40	40	0.002	32.35%	XP_015749603.1
Select seq ref|XP_015753714.1	PREDICTED: unconventional myosin-X-like [Acropora digitifera]	157	157	4.00E-41	28.42%	XP_015753714.1

**Spot 24.**	**GFP-like fluorescent chromoprotein cFP484 [Orbicella**	**XP_020605513.1**	**PEPTIDE SEQUENCES:**		**yprdiadyfkqsf**	
	Description	Total Score	Query	E value	Per. Ident	Accession
Select seq ref|XP_020605513.1	GFP-like fluorescent chromoprotein cFP484 [Orbicella faveolat	467	100%	1.00E-166	100.00%	XP_020605513.1
Select seq gb|ACD13193.1|	green fluorescent GFP-like protein [Favites abdita]	420	100%	5.00E-148	87.95%	ACD13193.1
Select seq gb|ABS87212.1|	green fluorescent protein [Montastraea cavernosa]	837	100%	3.00E-147	88.11%	ABS87212.1
Select seq gb|ABB17968.1|	green fluorescent GFP-like protein [Echinophyllia echinata]	417	100%	6.00E-147	87.50%	ABB17968.1
Select seq gb|AAO61601.1|	green fluorescent protein [Montastraea cavernosa]	417	100%	7.00E-147	87.67%	AAO61601.1
Select seq gb|AAO64989.1|	green fluorescent protein R4 [synthetic construct]	417	100%	1.00E-146	88.11%	AAO64989.1
Select seq gb|AAK83923.1|	green fluorescent protein [Orbicella faveolata]	417	100%	1.00E-146	88.11%	AAK83923.1
Select seq gb|AAO64994.1|	green fluorescent protein G4 [synthetic construct]	417	100%	1.00E-146	88.11%	AAO64994.1
Select seq gb|AAU04449.1|	green fluorescent protein G1 [Orbicella faveolata]	416	100%	3.00E-146	87.67%	AAU04449.1
Select seq gb|AAO43180.1|	green fluorescent protein [Monster GFP vector phMGFP]	414	99%	1.00E-145	87.61%	AAO43180.1
Select seq gb|AAV66356.1|	'Red/Green ancestor' ancestral fluorescent protein variant C05	414	100%	3.00E-145	87.05%	AAV66356.1

**Spot 25.**	**GPI inositol-deacylase-like [Orbicella faveolata]**	**XP_020615720.1**	**PEPTIDE SEQUENCES:**		**lygegeyadrskglrlsgip**	
	Description	Total Score	Query	E value	Per. Ident	Accession
Select seq ref|XP_020615720.1	GPI inositol-deacylase-like [Orbicella faveolata]	1811	100%	0	100.00%	XP_020615720.1
Select seq ref|XP_020616369.1	GPI inositol-deacylase-like [Orbicella faveolata]	1797	100%	0	98.98%	XP_020616369.1
Select seq ref|XP_027038149.1	GPI inositol-deacylase-like [Pocillopora damicornis]	1229	99%	0	68.71%	XP_027038149.1
Select seq ref|XP_022805945.1	GPI inositol-deacylase-like isoform X1 [Stylophora pistillata]	1207	100%	0	67.27%	XP_022805945.1
Select seq ref|XP_015778864.1	PREDICTED: GPI inositol-deacylase-like [Acropora digitifera]	1193	100%	0	64.07%	XP_015778864.1
Select seq gb|RMX50689.1|	hypothetical protein pdam_00013226 [Pocillopora damicornis]	1169	94%	0	68.62%	RMX50689.1

Select seq gb|PFX15813.1|	GPI inositol-deacylase [Stylophora pistillata]	1093	88%	0	69.12%	PFX15813.1
Select seq ref|XP_022805946.1	GPI inositol-deacylase-like isoform X2 [Stylophora pistillata]	959	81%	0	64.69%	XP_022805946.1
Select seq ref|XP_020907447.1	GPI inositol-deacylase [Exaiptasia pallida]	818	99%	0	45.02%	XP_020907447.1
Select seq ref|XP_001637866.1	predicted protein [Nematostella vectensis]	602	64%	0	51.40%	XP_001637866.1
Select seq ref|XP_002600834.1	hypothetical protein BRAFLDRAFT_279100 [Branchiostoma flo	498	96%	6.00E-159	33.98%	XP_002600834.1

**Spot 26.**	**Green fluorescent protein**	**ABC68475.1**	**PEPTIDE SEQUENCES:**		**dlkvvdggplpf**	
	Description	Total Score	Query Cover	E value	Per. Ident	Accession
Select seq gb|ABC68475.1|	green fluorescent protein [Orbicella faveolata]	469	100%	7.00E-171	100.00%	ABC68475.1
Select seq ref|XP_020605513.1|	GFP-like fluorescent chromoprotein cFP484 [Orbicella faveolat	399	100%	3.00E-143	84.89%	XP_020605513.1

Appendix A.3 (continued)

Select seq ref|XP_020605512.1|	GFP-like fluorescent chromoprotein cFP484 [Orbicella faveolat	397	100%	2.00E-142	83.84%	XP_020605512.1
Select seq gb|AAU04449.1|	green fluorescent protein G1 [Orbicella faveolata]	397	100%	3.00E-142	84.21%	AAU04449.1
Select seq gb|AAK83923.1|	green fluorescent protein [Orbicella faveolata]	394	100%	3.00E-141	83.77%	AAK83923.1
Select seq ref|XP_020623141.1|	GFP-like fluorescent chromoprotein cFP484 [Orbicella faveolat	394	100%	3.00E-141	82.53%	XP_020623141.1
Select seq ref|XP_020605521.1|	GFP-like fluorescent chromoprotein cFP484 isoform X1 [Orbice	387	100%	2.00E-138	81.22%	XP_020605521.1
Select seq ref|XP_020630220.1|	GFP-like fluorescent chromoprotein cFP484 [Orbicella faveolat	340	95%	3.00E-120	77.21%	XP_020630220.1
Select seq ref|XP_020630140.1|	GFP-like fluorescent chromoprotein cFP484 [Orbicella faveolat	307	99%	7.00E-107	65.64%	XP_020630140.1
Select seq ref|XP_020627712.1|	GFP-like fluorescent chromoprotein cFP484 [Orbicella faveolat	291	80%	3.00E-101	75.96%	XP_020627712.1
Select seq ref|XP_020630143.1|	GFP-like fluorescent chromoprotein cFP484 [Orbicella faveolat	580	96%	3.00E-100	63.47%	XP_020630143.1
Select seq ref|XP_020630180.1|	GFP-like fluorescent chromoprotein cFP484 [Orbicella faveolat	275	76%	5.00E-95	73.91%	XP_020630180.1

**Spot 27.**	**Pax C**	**B6DC09**	**PEPTIDE SEQUENCES:**		**kshypdvatreela**	
	Description	Total score	E value	Ident	Accession	
paired box protein Pax-3-B-like	75.5	100%	2.00E-18	92.50%	XP_020628638.1	
Select seq ref|XP_020630062.1	paired box protein Pax-3-B-like [Orbicella faveolata]	63.5	97%	3.00E-14	71.79%	XP_020630062.1
Select seq ref|XP_020631883.1	retinal homeobox protein Rx-B-like [Orbicella faveolata]	62	95%	5.00E-14	76.32%	XP_020631883.1
Select seq ref|XP_020603228.1	paired box protein Pax-6-like isoform X1 [Orbicella faveolata]	62.8	97%	6.00E-14	66.67%	XP_020603228.1
Select seq ref|XP_020629450.1	aristaless-related homeobox protein-like [Orbicella faveolata]	62.4	95%	8.00E-14	73.68%	XP_020629450.1
Select seq ref|XP_020632255.1	homeobox protein goosecoid-like [Orbicella faveolata]	59.3	97%	4.00E-13	66.67%	XP_020632255.1
Select seq ref|XP_020630078.1	paired box protein Pax-3-like [Orbicella faveolata]	58.2	97%	2.00E-12	64.10%	XP_020630078.1
Select seq ref|XP_020611047.1	homeobox protein unc-4 homolog [Orbicella faveolata]	58.2	100%	2.00E-12	65.00%	XP_020611047.1
Select seq ref|XP_020625918.1	paired box protein Pax-3-B-like [Orbicella faveolata]	57.8	100%	4.00E-12	62.50%	XP_020625918.1
Select seq ref|XP_020620522.1	ALX homeobox protein 1-like [Orbicella faveolata]	57.4	95%	5.00E-12	65.79%	XP_020620522.1
Select seq ref|XP_020628711.1	diencephalon/mesencephalon homeobox protein 1-A-like [Orb	56.6	97%	6.00E-12	56.41%	XP_020628711.1
Select seq ref|XP_020628665.1	paired mesoderm homeobox protein 2-like [Orbicella faveolata	56.2	97%	6.00E-12	58.97%	XP_020628665.1

**Spot 28.**	**BMP2/4, partial [*Orbicella faveolata*]**	**AHZ61804**	**PEPTIDE SEQUENCES:**		**vappgykafy**	
	Description	Total Score	Query	E value	Per. Ident	Accession

Select seq gb|AHZ61804.1|	BMP2/4 [Orbicella faveolata]	99	100%	3.00E-26	100.00%	AHZ61804.1
Select seq gb|AHZ61802.1|	BMP2/4 [Astrea curta]	97.4	100%	1.00E-25	97.83%	AHZ61802.1
Select seq gb|AHZ61765.1|	BMP2/4 [Dichocoenia stokesii]	97.4	100%	1.00E-25	97.83%	AHZ61765.1
Select seq gb|AHZ61740.1|	BMP2/4 [Acanthastrea echinata]	96.7	100%	2.00E-25	95.65%	AHZ61740.1
Select seq gb|AHZ61789.1|	BMP2/4 [Isophyllia sinuosa]	95.9	100%	4.00E-25	95.65%	AHZ61789.1
Select seq gb|AHZ61761.1|	BMP2/4 [Dendrogyra cylindrus]	95.5	100%	6.00E-25	95.65%	AHZ61761.1
Select seq gb|AHZ61769.1|	BMP2/4 [Dichocoenia stokesii]	95.5	100%	7.00E-25	95.65%	AHZ61769.1
Select seq gb|AHZ61773.1|	BMP2/4 [Pseudodiploria strigosa]	95.1	100%	8.00E-25	93.48%	AHZ61773.1
Select seq gb|AHZ61757.1|	BMP2/4 [Coscinaraea monile]	95.1	100%	8.00E-25	95.65%	AHZ61757.1
Select seq gb|AHZ61794.1|	BMP2/4 [Lobophyllia hemprichii]	95.1	100%	9.00E-25	93.48%	AHZ61794.1

Appendix A.3 (continued)

Select seq gb|AHZ61815.1|	BMP2/4 [Sandalolitha robusta]	94	100%	2.00E-24	93.48%	AHZ61815.1

**Spot 29.**	**Caspase 8**	**ADG23096.1**	**PEPTIDE SEQUENCES:**		**adlpygieeriqn**	
	Description	Max Score	Total Score	E value	Per. Ident	Accession
Select seq gb|ADG23096.1|	caspase 8 [Acropora palmata]	944	944	0	100.00%	ADG23096.1
Select seq ref|XP_015756325.1	PREDICTED: caspase-10-like [Acropora digitifera]	619	712	0	92.84%	XP_015756325.1
Select seq gb|AJG36593.1|	caspase 8 [Acropora millepora]	616	848	0	92.54%	AJG36593.1
Select seq gb|AJG36591.1|	caspase 8 [Acropora millepora]	615	848	0	92.54%	AJG36591.1
Select seq ref|XP_015756331.1	PREDICTED: caspase-3-like [Acropora digitifera]	416	644	5.00E-143	86.83%	XP_015756331.1
Select seq ref|XP_015758750.1	PREDICTED: LOW QUALITY PROTEIN: dynein heavy chain 2, axc	29.6	29.6	6.3	55.56%	XP_015758750.1
Select seq ref|XP_015780003.1	PREDICTED: volume-regulated anion channel subunit LRRC8A-l	71.2	71.2	6.00E-13	45.12%	XP_015780003.1
Select seq ref|XP_015780009.1	PREDICTED: volume-regulated anion channel subunit LRRC8A-l	72	72	4.00E-13	44.74%	XP_015780009.1
Select seq ref|XP_015768208.1	PREDICTED: caspase-3-like [Acropora digitifera]	39.3	39.3	0.005	42.37%	XP_015768208.1
Select seq ref|XP_015766400.1	PREDICTED: caspase-3-like [Acropora digitifera]	89.4	89.4	3.00E-19	36.69%	XP_015766400.1
Select seq ref|XP_015774399.1	PREDICTED: uncharacterized protein LOC107352599 [Acropora	57.4	57.4	1.00E-08	36.59%	XP_015774399.1
Select seq ref|XP_015770993.1	PREDICTED: uncharacterized protein LOC107349364 isoform X	60.5	60.5	1.00E-09	34.78%	XP_015770993.1
Select seq ref|XP_015770992.1	PREDICTED: uncharacterized protein LOC107349364 isoform X	60.5	60.5	1.00E-09	34.78%	XP_015770992.1

**Spot 30.**	**Ectonucleoside triphosphate diphosphohydrolase 1**	**XP_015779265**	**PEPTIDE SEQUENCES:**		**gdiikgtalvkqi**	
	Description	Max Score	Total Score	E value	Per. Ident	Accession
Select seq ref|XP_015779265.1	PREDICTED: ectonucleoside triphosphate diphosphohydrolase	596	596	0	100.00%	XP_015779265.1
Select seq ref|XP_015756595.1	PREDICTED: nucleoside-triphosphatase ntp-1-like [Acropora di	111	111	5.00E-29	33.90%	XP_015756595.1
Select seq ref|XP_015756628.1	PREDICTED: ectonucleoside triphosphate diphosphohydrolase	94	94	1.00E-21	32.33%	XP_015756628.1
Select seq ref|XP_015768859.1	PREDICTED: ectonucleoside triphosphate diphosphohydrolase	93.2	93.2	1.00E-21	33.90%	XP_015768859.1
Select seq ref|XP_015768456.1	PREDICTED: ectonucleoside triphosphate diphosphohydrolase	84	84	1.00E-18	33.68%	XP_015768456.1

**Spot 31.**	**Carbonic anhydrase 5A, mitochondrial-like [Orbicella**	**XP_020613891.1**	**PEPTIDE SEQUENCES:**		**laqflktvrhl**	
	Description	Total Score	Query	E value	Per. Ident	Accession

Select seq ref|XP_020613615.1	carbonic anhydrase 2-like [Orbicella faveolata]	229	86%	2.00E-74	92.50%	XP_020613615.1
Select seq ref|XP_022803955.1	carbonic anhydrase 1-like [Stylophora pistillata]	171	71%	1.00E-49	79.59%	XP_022803955.1
Select seq ref|XP_027050584.1	carbonic anhydrase 1-like [Pocillopora damicornis]	169	71%	6.00E-49	77.55%	XP_027050584.1
Select seq ref|XP_029205258.1	uncharacterized protein LOC114969126 [Acropora millepora]	161	78%	3.00E-43	66.97%	XP_029205258.1
Select seq ref|XP_015770689.1	PREDICTED: uncharacterized protein LOC107349103 [Acropora	160	78%	5.00E-43	66.06%	XP_015770689.1
Select seq ref|XP_020901977.1	carbonic anhydrase 7 [Exaiptasia pallida]	141	72%	1.00E-38	62.00%	XP_020901977.1
Select seq gb|ANJ59782.1|	alpha carbonic anhydrase 6 [Exaiptasia pallida]	140	72%	2.00E-38	62.00%	ANJ59782.1
Select seq ref|XP_001632501.1	predicted protein [Nematostella vectensis]	131	69%	3.00E-35	60.82%	XP_001632501.1
Select seq gb|ODN05569.1|	Carbonic anhydrase 2 [Orchesella cincta]	101	65%	3.00E-23	51.65%	ODN05569.1
Select seq ref|XP_020600771.1	carbonic anhydrase-like isoform X2 [Orbicella faveolata]	100	67%	2.00E-22	48.39%	XP_020600771.1
Select seq ref|XP_020600769.1	carbonic anhydrase-like isoform X1 [Orbicella faveolata]	100	67%	5.00E-22	48.39%	XP_020600769.1
Select seq gb|PRD34444.1|	Ca13 [Trichonephila clavipes]	91.7	74%	1.00E-20	45.63%	PRD34444.1

Appendix A.3 (continued)

Spot 32.	Calmodullin	ACY07618.1	PEPTIDE SEQUENCES:		vmrncgesltnae	
	Description	Max score	Total score	E value	Ident	Accession
Select seq gb|ACY07618.1|	calmodulin-like protein [Acropora millepora]	16.8	25.9	26	67%	ACY07618.1
Select seq dbj|BAQ19096.1|	paired box B protein [Acropora digitifera]	15.9	15.9	54	100%	BAQ19096.1
Select seq gb|AAF64460.1|AF2	transcription factor PaxB [Acropora millepora]	15.9	15.9	54	100%	AAF64460.1
Select seq sp|B3EWZ3.1|CADN	RecName: Full=Coadhesin	15.9	47.7	54	100%	B3EWZ3.1
Select seq sp|B3EWZ5.1|MLRP	RecName: Full=MAM and LDL-receptor class A domain-contain	15.9	31.8	54	100%	B3EWZ5.1
Select seq sp|B3EWZ6.1|MLRP	RecName: Full=MAM and LDL-receptor class A domain-contain	15.9	25.9	54	100%	B3EWZ6.1
Select seq gb|KJD18916.1|	hypothetical protein VE30_10855 [Halomonas meridiana]	15.5	15.5	79	67%	KJD18916.1
Select seq gb|AFI99104.1|	56kDa selenium binding protein [Acropora millepora]	15.5	15.5	79	80%	AFI99104.1
Select seq sp|B3EWY9.1|MLP_	RecName: Full=Mucin-like protein	15.5	29.7	79	71%	B3EWY9.1
Select seq gb|AET09738.1|	hypothetical protein p4_37 [Acropora millepora]	15.1	15.1	116	100%	AET09738.1
Select seq gb|ADG44981.1|	hypothetical protein [Acropora millepora]	15.1	15.1	116	100%	ADG44981.1
Select seq gb|KJD20578.1|	spermidine/putrescine ABC transporter substrate-binding prot	15.1	15.1	116	100%	KJD20578.1
Select seq dbj|BAQ19100.1|	orthodenticle homeobox A protein [Acropora digitifera]	13.4	13.4	531	80%	BAQ19100.1
Select seq gb|ABK41270.1|	orthodenticle A [Acropora millepora]	13.4	13.4	531	80%	ABK41270.1
Select seq sp|B8RJM0.1|USOM	RecName: Full=Uncharacterized skeletal organic matrix proteir	12.5	12.5	1128	60%	B8RJM0.1

Spot 33.	Scleractinian cysteine-rich protein [*Montastraea faveolata*]	B2ZG38	PEPTIDE SEQUENCES:		aakfhlclll	
	Description	Total Score	Query	E value	Per. Ident	Accession
Select seq sp|B2ZG38.1|	RecName: Full=Small cysteine-rich protein 8; Short=Mfav-SCRiI	153	100%	6.00E-47	100.00%	B2ZG38.1
Select seq sp|C0H693.1|	RecName: Full=Small cysteine-rich protein 1 1; Short=Mcap-SC	51.2	97%	1.00E-06	36.25%	C0H693.1
Select seq sp|C0H694.1|	RecName: Full=Small cysteine-rich protein 1 2; Short=Mcap-SC	50.8	97%	2.00E-06	36.25%	C0H694.1
Select seq ref|XP_020613205.1	small cysteine-rich protein 8-like [Orbicella faveolata]	48.1	98%	3.00E-05	53.57%	XP_020613205.1

Select seq ref|XP_015765734.1	PREDICTED: small cysteine-rich protein 1 2-like isoform X1 [Acr	40.8	89%	0.026	32.00%	XP_015765734.1
Select seq ref|XP_020631985.1	small cysteine-rich protein 6 [Orbicella faveolata]	37	63%	0.59	48.00%	XP_020631985.1
Select seq ref|XP_020610104.1	small cysteine-rich protein 6-like [Orbicella faveolata]	36.6	87%	0.64	42.86%	XP_020610104.1
Select seq ref|XP_020610088.1	small cysteine-rich protein 6-like [Orbicella faveolata]	36.6	63%	1.3	46.00%	XP_020610088.1
Select seq ref|WP_126197983.:	oligopeptide ABC transporter ATP-binding protein OppD [Serra	37.4	44%	1.3	51.52%	WP_126197983.1
Select seq ref|NP_536705.3|	mucin-4 precursor [Mus musculus]	37.4	51%	1.3	38.64%	NP_536705.3
Select seq ref|WP_126199601.:	oligopeptide ABC transporter ATP-binding protein OppD [Serra	37.4	44%	1.4	51.52%	WP_126199601.1
Select seq ref|XP_021041269.1	mucin-4 [Mus caroli]	37.4	51%	1.7	38.64%	XP_021041269.1
Select seq ref|WP_079656102.:	dipeptide ABC transporter ATP-binding protein [Serratia marce	37	44%	1.9	51.52%	WP_079656102.1

Appendix A.3 (continued)

Spot 34.	DnaJ homolog subfamily C member 5-like [Orbicella	XP_020605856.1	PEPTIDE SEQUENCES:		malkhhpdkn	
	Description	Total Score	Query	E value	Per. Ident	Accession
Select seq ref|XP_020605856.1	dnaJ homolog subfamily C member 5-like [Orbicella faveolata]	153	100%	7.00E-51	100.00%	XP_020605856.1
Select seq ref|XP_020629051.1	dnaJ homolog subfamily B member 2-like [Orbicella faveolata]	56.2	63%	3.00E-11	57.45%	XP_020629051.1
Select seq ref|XP_020614854.1	dnaJ homolog subfamily A member 2-like [Orbicella faveolata]	55.8	65%	6.00E-11	53.19%	XP_020614854.1
Select seq ref|XP_020626187.1	dnaJ homolog subfamily B member 11-like [Orbicella faveolata	55.5	75%	1.00E-10	44.44%	XP_020626187.1
Select seq ref|XP_020622054.1	dnaJ protein homolog 1-like [Orbicella faveolata]	53.9	86%	3.00E-10	45.16%	XP_020622054.1
Select seq ref|XP_020606156.1	dnaJ homolog subfamily B member 6-like [Orbicella faveolata]	53.1	59%	4.00E-10	56.82%	XP_020606156.1
Select seq ref|XP_020622055.1	dnaJ homolog subfamily B member 1-like [Orbicella faveolata]	53.5	73%	4.00E-10	49.06%	XP_020622055.1
Select seq ref|XP_020618047.1	dnaJ homolog subfamily A member 1-like [Orbicella faveolata]	52.8	65%	7.00E-10	51.06%	XP_020618047.1

Spot 35.	Alpha i G protein	AFZ78088.1	PEPTIDE SEQUENCES:		caqyrqvvy	
	Description	Max Score	Total Score	E value	Per. Ident	Accession
Select seq gb|AFZ78088.1|	alpha i G protein [Acropora palmata]	741	741	0	100.00%	AFZ78088.1
Select seq ref|XP_015777008.1	PREDICTED: guanine nucleotide-binding protein G(o) subunit a	496	496	1.00E-177	66.95%	XP_015777008.1
Select seq ref|XP_015780306.1	PREDICTED: guanine nucleotide-binding protein G(q) subunit a	328	328	2.00E-111	49.15%	XP_015780306.1
Select seq ref|XP_015772099.1	PREDICTED: guanine nucleotide-binding protein subunit alpha-	298	298	3.00E-99	42.49%	XP_015772099.1
Select seq gb|AFZ78087.1|	alpha c G protein [Acropora palmata]	269	269	7.00E-88	38.59%	AFZ78087.1
Select seq ref|XP_015764888.1	PREDICTED: guanine nucleotide-binding protein G(i) subunit al	207	207	2.00E-67	100.00%	XP_015764888.1
Select seq ref|XP_015770327.1	PREDICTED: guanine nucleotide-binding protein G(s) subunit al	213	213	6.00E-66	36.71%	XP_015770327.1
Select seq ref|XP_015768491.1	PREDICTED: guanine nucleotide-binding protein G(t) subunit al	88.2	88.2	8.00E-21	34.90%	XP_015768491.1
Select seq ref|XP_015779045.1	PREDICTED: ADP-ribosylation factor 1 isoform X2 [Acropora dig	50.1	50.1	3.00E-07	29.00%	XP_015779045.1
Select seq ref|XP_015779044.1	PREDICTED: ADP-ribosylation factor 1 isoform X1 [Acropora dig	48.5	48.5	1.00E-06	29.00%	XP_015779044.1
Select seq ref|XP_020632509.1	dnaJ homolog subfamily B member 13-like [Orbicella faveolata	51.6	65%	2.00E-09	53.19%	XP_020632509.1

Select seq ref|XP_020609046.1	dnaJ homolog subfamily B member 9-like [Orbicella faveolata]	47.4	65%	6.00E-08	44.68%	XP_020609046.1
Select seq ref|XP_020625391.1	dnaJ homolog subfamily B member 14-like [Orbicella faveolata	46.2	59%	2.00E-07	48.84%	XP_020625391.1
Select seq ref|XP_020603256.1	dnaJ homolog subfamily C member 21-like [Orbicella faveolata	45.4	58%	3.00E-07	51.16%	XP_020603256.1
Select seq ref|XP_020618202.1	dnaJ homolog subfamily C member 17-like [Orbicella faveolata	44.7	54%	6.00E-07	51.28%	XP_020618202.1
Select seq ref|XP_020617012.1	dnaJ homolog subfamily C member 11-like [Orbicella faveolata	44.3	65%	9.00E-07	42.00%	XP_020617012.1

Spot 36. Small cysteine-rich protein 6 C1KIZ5 PEPTIDE SEQUENCES: iilgaltvqg avsg

	Description	Max Score	Total Score	E value	Per. Ident	Accession
Select seq ref|XP_020631985.1	small cysteine-rich protein 6 [Orbicella faveolata]	169	169	2.00E-53	100.00%	XP_020631985.1
Select seq ref|XP_020610088.1	small cysteine-rich protein 6-like [Orbicella faveolata]	129	129	7.00E-37	76.54%	XP_020610088.1
Select seq ref|XP_020610104.1	small cysteine-rich protein 6-like [Orbicella faveolata]	86.7	86.7	2.00E-20	61.54%	XP_020610104.1
Select seq sp|C1KIZ3.1|	RecName: Full=Small cysteine-rich protein 4; Short=Mfav-SCRiI	84.3	84.3	2.00E-19	63.29%	C1KIZ3.1
Select seq ref|XP_022817917.1	uncharacterized protein LOC111350529 [Spodoptera litura]	39.7	39.7	0.27	37.04%	XP_022817917.1

Appendix A.3 (continued)

Spot 37. Zinc finger protein 768-like [Orbicella faveolata] XP_020615964.1 PEPTIDE SEQUENCES: gytvdflpypvse

	Description	Max Score	Total Score	E value	Per. Ident	Accession
Select seq gb|AHA90941.1|	cytochrome oxidase subunit I [Orbicella faveolata]	245	245	100%	100.00%	AHA90940.1
Select seq gb|AHA90942.1|	cytochrome oxidase subunit I [Orbicella faveolata]	245	245	5.00E-81	99.20%	AHA90942.1
Select seq gb|AHA90943.1|	cytochrome oxidase subunit I [Orbicella faveolata]	243	243	6.00E-81	99.20%	AHA90943.1
Select seq gb|AHA90945.1|	cytochrome oxidase subunit I [Orbicella faveolata]	244	244	6.00E-81	99.20%	AHA90945.1
Select seq gb|AHA90941.1|	cytochrome oxidase subunit I [Orbicella faveolata]	244	244	8.00E-81	99.20%	AHA90941.1
Select seq gb|AFP73387.1|	cytochrome oxidase subunit I [Pseudodiploria strigosa]	243	243	4.00E-80	97.60%	AFP73387.1
Select seq gb|AAS17027.1|	cytochrome oxidase subunit I [Orbicella annularis]	244	244	4.00E-80	99.20%	AAS17027.1
Select seq gb|ACJ09533.1|	cytochrome oxidase subunit I [Platygyra pini]	242	242	6.00E-80	98.40%	ACJ09533.1
Select seq gb|ABY85346.1|	cytochrome oxidase subunit I [Favites cf. valenciennesi DH-201	243	243	6.00E-80	98.40%	ABY85346.1
Select seq gb|ABY85345.1|	cytochrome oxidase subunit I [Favites cf. valenciennesi DH-201	243	243	7.00E-80	98.40%	ABY85345.1
Select seq dbj|BAD11414.1|	cytochrome oxidase I [Orbicella annularis]	243	243	9.00E-80	99.20%	BAD11414.1
Select seq gb|ABY85307.1|	cytochrome oxidase subunit I [Dipsastraea matthaii]	242	242	1.00E-79	98.40%	ABY85307.1

Spot 38. Alpha q G protein AFZ78090 PEPTIDE SEQUENCES: ndegmkecyd

	Description	Max Score	Total Score	E value	Per. Ident	Accession
Select seq gb|AFZ78090.1|	alpha q G protein [Acropora palmata]	736	736	0	100.00%	AFZ78090.1
Select seq ref|XP_015780306.1	PREDICTED: guanine nucleotide-binding protein G(q) subunit a	733	733	0	99.43%	XP_015780306.1
Select seq ref|XP_015777008.1	PREDICTED: guanine nucleotide-binding protein G(o) subunit a	333	333	4.00E-113	49.72%	XP_015777008.1
Select seq gb|AFZ78089.1|	alpha o G protein [Acropora palmata]	329	329	9.00E-112	49.44%	AFZ78089.1
Select seq gb|AFZ78088.1|	alpha i G protein [Acropora palmata]	329	329	1.00E-111	49.15%	AFZ78088.1

Select seq ref|XP_015772099.1	PREDICTED: guanine nucleotide-binding protein subunit alpha-	348	348	2.00E-119	49.15%	XP_015772099.1
Select seq ref|XP_015764888.1	PREDICTED: guanine nucleotide-binding protein G(i) subunit al	81.3	81.3	7.00E-19	43.16%	XP_015764888.1
Select seq ref|XP_015770327.1	PREDICTED: guanine nucleotide-binding protein G(s) subunit al	233	233	2.00E-73	37.47%	XP_015770327.1
Select seq gb|AFZ78087.1|	alpha c G protein [Acropora palmata]	226	226	2.00E-71	35.40%	AFZ78087.1
Select seq ref|XP_015768491.1	PREDICTED: guanine nucleotide-binding protein G(t) subunit al	71.2	71.2	9.00E-15	29.58%	XP_015768491.1
Select seq ref|XP_015759852.1	PREDICTED: ADP-ribosylation factor 3-like [Acropora digitifera]	46.2	46.2	8.00E-06	27.84%	XP_015759852.1
Select seq ref|XP_015779044.1	PREDICTED: ADP-ribosylation factor 1 isoform X1 [Acropora dig	41.6	41.6	2.00E-04	26.60%	XP_015779044.1
Select seq ref|XP_015779045.1	PREDICTED: ADP-ribosylation factor 1 isoform X2 [Acropora dig	40	40	7.00E-04	26.60%	XP_015779045.1

Spot 39. **Alpha carbonic anhydrase** **AHZ61699.1** **PEPTIDE SEQUENCES:** **nisggglsqfraa**

	Description	Max Score	Total Score	E value	Per. Ident	Accession
Select seq ref|XP_015757476.1	PREDICTED: pancreatic secretory granule membrane major gly	25.4	25.4	8.9	88.89%	XP_015757476.1
Select seq ref|XP_015757475.1	PREDICTED: multiple epidermal growth factor-like domains prc	25.4	25.4	9.9	72.73%	XP_015757475.1
Select seq gb|AHZ61629.1|	alpha carbonic anhydrase [Acropora cervicornis]	155	155	1.00E-50	71.00%	AHZ61629.1

Appendix A.3 (continued)

Select seq gb|AHZ61634.1|	alpha carbonic anhydrase [Acropora tenuis]	145	145	4.00E-47	67.33%	AHZ61634.1
Select seq ref|XP_015774933.1	PREDICTED: carbonic anhydrase 1-like isoform X1 [Acropora di	67	67	5.00E-15	46.43%	XP_015774933.1
Select seq ref|XP_015761724.1	PREDICTED: putative carbonic anhydrase [Acropora digitifera]	40.8	40.8	3.00E-05	46.15%	XP_015761724.1
Select seq ref|XP_015760670.1	PREDICTED: low-density lipoprotein receptor-related protein 4	27.7	27.7	1.8	42.42%	XP_015760670.1
Select seq gb|ACJ64663.1|	hypothetical protein A030-E11 [Acropora millepora]	85.5	85.5	1.00E-21	41.67%	ACJ64663.1
Select seq ref|XP_015771455.1	PREDICTED: carbonic anhydrase 2-like [Acropora digitifera]	85.5	85.5	1.00E-21	41.67%	XP_015771455.1
Select seq ref|XP_015771376.1	PREDICTED: carbonic anhydrase 12-like [Acropora digitifera]	71.2	129	8.00E-16	41.10%	XP_015771376.1
Select seq ref|XP_015759239.1	PREDICTED: putative carbonic anhydrase-like protein 2 [Acropo	65.9	65.9	5.00E-14	41.10%	XP_015759239.1
Select seq ref|XP_015759705.1	PREDICTED: carbonic anhydrase 2-like [Acropora digitifera]	77.4	77.4	3.00E-18	38.24%	XP_015759705.1
Select seq ref|XP_015774481.1	PREDICTED: putative carbonic anhydrase [Acropora digitifera]	45.8	45.8	5.00E-07	36.36%	XP_015774481.1
Select seq gb|ACJ64662.1|	hypothetical protein C007-E7 [Acropora millepora]	69.7	69.7	2.00E-15	35.64%	ACJ64662.1
Select seq ref|XP_015750571.1	PREDICTED: putative carbonic anhydrase [Acropora digitifera]	47	47	2.00E-07	34.33%	XP_015750571.1
Select seq ref|XP_015770689.1	PREDICTED: uncharacterized protein LOC107349103 [Acropora	69.7	69.7	3.00E-15	30.93%	XP_015770689.1

Spot 40. **alpha carbonic anhydrase [*Montastraea faveolata*]** **XP_020628594.1** **PEPTIDE SEQUENCES:** **kdyrdlkiaf**

	Description	Total Score	Query	E value	Per. Ident	Accession
Select seq ref|XP_020628594.1	uncharacterized protein LOC110065766 [Orbicella faveolata]	863	863	100%	0	XP_020628594.1
Select seq ref|XP_020628593.1	carbonic anhydrase 2-like [Orbicella faveolata]	332	332	47%	9.00E-109	XP_020628593.1
Select seq ref|XP_027055172.1	carbonic anhydrase-related protein 10-like [Pocillopora damicc	211	374	50%	8.00E-60	XP_027055172.1
Select seq gb|RMX36692.1|	hypothetical protein pdam_00015839 [Pocillopora damicornis]	213	400	50%	1.00E-59	RMX36692.1
Select seq gb|ANJ59776.1|	alpha carbonic anhydrase 16 [Stylophora pistillata]	204	409	49%	3.00E-59	ANJ59776.1
Select seq ref|XP_022801331.1	carbonic anhydrase 2-like [Stylophora pistillata]	192	192	49%	1.00E-54	XP_022801331.1
Select seq gb|PFX18799.1|	Carbonic anhydrase 2 [Stylophora pistillata]	192	192	49%	2.00E-54	PFX18799.1
Select seq ref|XP_027050353.1	carbonic anhydrase 2-like [Pocillopora damicornis]	188	188	40%	2.00E-54	XP_027050353.1

Select seq ref|XP_022799897.1	N66 matrix protein-like [Stylophora pistillata]	180	242	45%	1.00E-48	XP_022799897.1
Select seq ref|XP_022801327.1	carbonic anhydrase 2-like [Stylophora pistillata]	171	171	35%	3.00E-47	XP_022801327.1
Select seq ref|XP_022801328.1	carbonic anhydrase 2-like [Stylophora pistillata]	169	169	35%	2.00E-46	XP_022801328.1
Select seq gb|PFX19740.1|	Carbonic anhydrase 2 [Stylophora pistillata]	181	461	50%	7.00E-46	PFX19740.1

**Spot 41.**	**Galaxin**	**D9IQ16.1**	**PEPTIDE SEQUENCES:**		**nveprvgaspmc**	
	Description	Total Score	Query	E value	Per. Ident	Accession
Select seq sp|D9IQ16.1|	RecName: Full=Galaxin; Flags: Precursor [Acropora millepora]	681	100%	0	100.00%	D9IQ16.1
Select seq ref|XP_029187982.1	galaxin [Acropora millepora]	676	100%	0	99.41%	XP_029187982.1
Select seq ref|XP_015751001.1	PREDICTED: galaxin [Acropora digitifera]	674	100%	0	98.82%	XP_015751001.1
Select seq gb|ARA71553.1|	galaxin [Acropora digitifera]	668	100%	0	97.93%	ARA71553.1
Select seq gb|ART34494.1|	galaxin [Galaxea astreata]	387	97%	3.00E-131	58.88%	ART34494.1
Select seq pir|JC7958|	galaxin precursor - reef coral [Galaxea fascicularis]	387	97%	6.00E-131	58.58%	JC7958
Select seq gb|ABV24967.1|	galaxin [Montipora capitata]	647	87%	4.00E-55	67.91%	ABV24967.1
Select seq gb|PFX30452.1|	Usherin [Stylophora pistillata]	1044	85%	6.00E-51	39.56%	PFX30452.1

Appendix A.3 (continued)

Select seq ref|XP_022782678.1	galaxin-like [Stylophora pistillata]	1038	85%	4.00E-50	38.79%	XP_022782678.1
Select seq ref|XP_015761791.1	PREDICTED: galaxin-like [Acropora digitifera]	472	83%	7.00E-47	35.56%	XP_015761791.1
Select seq gb|RMX54379.1|	hypothetical protein pdam_00021390 [Pocillopora damicornis]	722	86%	8.00E-44	34.34%	RMX54379.1
Select seq ref|XP_027050600.1	galaxin-like [Pocillopora damicornis]	718	82%	2.00E-43	36.61%	XP_027050600.1
Select seq gb|ELU11211.1|	hypothetical protein CAPTEDRAFT_64481 [Capitella teleta]	152	80%	4.00E-40	36.33%	ELU11211.1

**Spot 42.**	**Procollagen**	**XP_020604034**	**PEPTIDE SEQUENCES:**		**dhnedntsailq**	
	Description	Total Score	Query	E value	Per. Ident	Accession
Select seq ref|XP_020604034.1	procollagen galactosyltransferase 1-like [Orbicella faveolata]	1239	100%	0	100.00%	XP_020604034.1
Select seq ref|XP_027048049.1	procollagen galactosyltransferase 1-like [Pocillopora damicorni	1081	100%	0	86.36%	XP_027048049.1
Select seq ref|XP_015757920.1	PREDICTED: procollagen galactosyltransferase 1-like [Acropora	989	100%	0	80.98%	XP_015757920.1
Select seq ref|XP_029214257.1	procollagen galactosyltransferase 1-like [Acropora millepora]	988	100%	0	80.98%	XP_029214257.1
Select seq ref|XP_022783764.1	procollagen galactosyltransferase 1-like [Stylophora pistillata]	955	98%	0	79.05%	XP_022783764.1
Select seq ref|XP_001635452.1	predicted protein [Nematostella vectensis]	1499	91%	0	63.92%	XP_001635452.1
Select seq gb|PFX29736.1|	Procollagen galactosyltransferase 1 [Stylophora pistillata]	884	85%	0	80.83%	PFX29736.1
Select seq ref|XP_028394615.1	procollagen galactosyltransferase 1-like [Dendronephthya giga	586	100%	0	48.24%	XP_028394615.1
Select seq ref|XP_027404007.1	procollagen galactosyltransferase 1 [Bos indicus x Bos taurus]	576	90%	0	51.49%	XP_027404007.1
Select seq ref|NP_001092425.1	procollagen galactosyltransferase 1 precursor [Bos taurus]	576	90%	0	51.49%	NP_001092425.1
Select seq ref|XP_006057968.1	LOW QUALITY PROTEIN: procollagen galactosyltransferase 1 [B	575	90%	0	51.49%	XP_006057968.1
Select seq ref|XP_025313769.1	procollagen galactosyltransferase 1 [Canis lupus dingo]	575	90%	0	51.30%	XP_025313769.1
Select seq ref|XP_023989967.1	procollagen galactosyltransferase 1 [Physeter catodon]	574	90%	0	50.93%	XP_023989967.1
Select seq gb|OWK12026.1|	COLGALT1 [Cervus elaphus hippelaphus]	574	92%	0	50.64%	OWK12026.1
