# Supplementary figures and images for: Seasonal Proteome Variations in Orbicella faveolata Reveal Molecular Thermal Stress Adaptations
Source: Proteomes. 2024 Jul 10;12(3):20. doi: 10.3390/proteomes12030020 (PMC11270422; doi:10.3390/proteomes12030020)

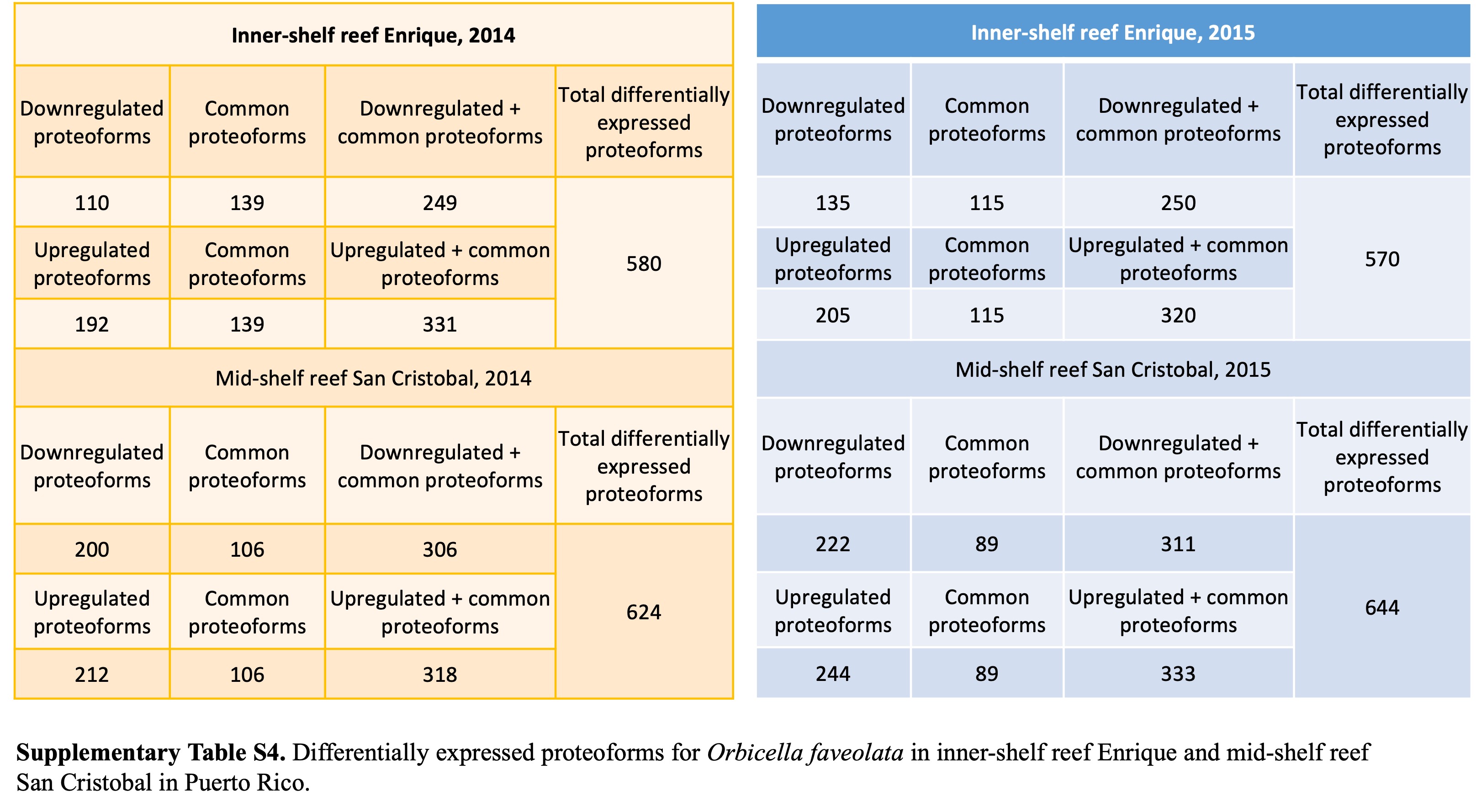

Supplement: Supplementary file 1 [file proteomes-12-00020-s001.zip › O.f. Supp. Table S4. 02.12.24.jpg]
